# Divergent functions of three Kunitz trypsin inhibitor (KTI) proteins in herbivore defense in poplar

**DOI:** 10.1186/s12870-025-07955-z

**Published:** 2025-12-19

**Authors:** Ishani S. Das, Qianqian Shi, Steven Dreischhoff, Andrea Polle

**Affiliations:** 1 Forest Botany and Tree Physiology, University of Göttingen, Büsgenweg 2, Göttingen, 37077 Germany; 2 Göttingen Center for Molecular Biosciences (GZMB), Justus-Von-Liebig-Weg 11, Göttingen, 37077 Germany; 3 Present Address: Present address: Institute for Plant Sciences, Cologne Biocenter, University of Cologne, Zülpicherstr. 47a, Cologne, 50674 Germany; 4 Present Address: Present address: College of Landscape Architecture and Arts, Northwest Agriculture & Forestry University, Yangling, Shaanxi 712100 China

**Keywords:** Jasmonic acid, Kunitz protease inhibitor, Phytohormone, CRISPR-Cas, Caterpillar, Lepidoptera, *Helicoverpa armigera*, *Populus*

## Abstract

**Background:**

Climate warming promotes the expansion of insect pests. Among the inducible defense responses activated by attacked plants, Kunitz trypsin protease inhibitors (KTIs) play an outstanding role. KTIs affect food digestion and thereby control the fitness of herbivorous insects. Poplars contain an expanded family of KTIs, whose distinct intrinsic functions are under investigation. Here, we set out to identify KTIs with anti-herbivore activity and assessed the potential growth trade-off incurred by high KTI expression levels.

**Results:**

Using in-silico database searches, we identified 28 KTIs in the haploid genome of *Populus* x *canescens*; 21 of them were responsive to herbivory. The greatest induction by herbivory was observed for *KTI_400, KTI_600* and *KTI_0882* (*P. trichocarpa* orthologues Potri.019G124400, Potri.019G124600, Potri.019G088200), whereas a moderate response was found for KTI_53200 (Potri.017G153200 orthologue). Mechanical wounding and methyl-jasmonate treatments resulted in fast and strong induction of *KTI_400* and *KTI_600* and moderate or lacking responses in *KTI_0882* and KTI_53200. Increased *KTI* expression levels were associated with upregulation of *ALLENE OXIDE SYNTHASE*, a key enzyme involved in jasmonate biosynthesis. On the contrary, exposure to compounds eliciting ethylene or salicylic acid signaling did not affect *KTI*s. We generated stable CRISPR-Cas12a-mediated knock-out and *p35S*-mediated overexpression lines of *KTI_400, KTI_600* and KTI_53200 in *Populus* x *canescens.* Among the wildtype and transgenic lines, only *kti_400* + *kti_600* double knock-out lines produced greater biomass. Larvae of *Helicoverpa armigera,* a pest expanding in Europe due to a warmer climate, were allowed to feed on wildtype and transgenic poplar lines. Transgenic poplars overexpressing *KTI_400* or *KTI_600* resulted in reduced, and their double knockout lines in increased weight gain of the larvae. In contrast, overexpressing or knockout lines of KTI_53200 had no effect on larval weight gain compared with controls.

**Conclusion:**

KTI_400 and KTI_600 are potent, natural *in-planta* anti-herbivorous agents. Their expression is associated with larval growth reductions. Modulation of *KTI_53200* levels had no direct effects on the fitness of leaf-feeding *H. armigera* or on plant growth. This study sheds light on the potential application of *KTI* in plant defenses and biocontrol against *H. armigera* in trees and presents new options to investigate growth-defense theories.

**Supplementary Information:**

The online version contains supplementary material available at 10.1186/s12870-025-07955-z.

## Background

Forests are facing an escalating array of anthropogenic-induced changes to their environment. Rising global temperatures, driven by human activities, have led to extended breeding seasons and increased generation cycles for insect herbivores [67]. Herbivory and pathogens pose significant threats to the growth and recruitment of poplars in northern biomes [67]. As keystone species, poplars play a crucial role in supporting associated organisms [70], and their industrial relevance as a feedstock for biofuels and bioproducts only adds to their importance [80]. Consequently, a deeper understanding of how poplars respond to and adapt to environmental cues is a pressing need.

Poplars possess a range of constitutive and inducible defenses, including metabolite- and protein-based mechanisms [22, 26]. Among these defenses, proteinase inhibitors are of particular interest due to their role in protecting against herbivory [34]. Proteinase inhibitors are widespread throughout the plant body [48, 49, 65]. When ingested by leaf-feeding herbivores, these proteins are absorbed and inhibit the activity of digestive enzymes in the insect's gut [67]. This inhibition leads to impaired food digestion, reduced dietary assimilation, and, in some cases, mortality of the herbivore [54, 61].

Protease inhibitors interact with different classes of proteases [71]. Kunitz Trypsin Inhibitor (KTI) proteins are part of the serine protease inhibitor family, known as “serpins”, and target serine proteases such as trypsin and chymotrypsin [71]. KTIs have a molecular mass of approximately 21 kDa, with approximately 120 amino acids and four cysteine residues. These cysteine residues form two disulfide bridges, which contribute to stabilize the reactive site. The reactive loop of KTIs with the P1-P1´ site binds to the active site of serine proteases [12]. Several mechanisms exist how proteinase inhibitors interact with their targets, resulting in bifunctional activities [34]. For example, they block alpha-amylases from barley grains [1] and inhibit proteases from the pathogenic fungus *Fusarium culmorum* [62]. In addition to mediating herbivore interactions [41, 49, 50, 53, 64, 65], KTIs are implicated in physiological functions such as nitrogen recycling during senescence [37] and protein processing during development [71].

The interaction of plants with insects is regulated by phytohormones of the jasmonate (JA) pathway [29, 47]. Oral secretions of insects contain elicitors like volicitin, inceptin, and caeliferins, which can induce JA-specific responses [2, 73, 75]. Furthermore, JAs (jasmonate-derivatives) are produced in response to wounding [20, 31, 68]. Feeding of gypsy moths on poplar leaves and treatment with JA cause partially overlapping responses, including *KTI* induction [6]. However, insect feeding also induces other signaling pathways such as ethylene or abscisic acid (ABA) signaling, in parallel with KTIs [6, 41].

In poplar, *KTI*s form a large gene family with more than 30 members [13, 28, 35, 49, 65]. KTIs occur in roots, leaves, bark and xylem sap [35, 43]. Transcriptomic analysis revealed that specific *KTIs* of *P. trichocarpa* x *deltoides* show distinct profiles across different tissue types impacted by herbivore attack (*Malacosoma disstria*) [65]. Ectopic overexpression of two distinct poplar *KTI* genes in *Arabidopsis* conferred varying levels of resistance against *Plutella xylostella* [17], suggesting functional divergence among KTI family members. It was also observed that *KTI*s in *P. nigra* are transcriptionally regulated in a herbivore-specific manner [28]. Due to the wide variety of transcriptional responses observed, it is not surprising that the specific functions of most KTI family members in poplar's defense against herbivory have not yet been experimentally investigated. This knowledge gap must be addressed to gain a clearer understanding of how defenses are regulated in poplar.

This study aimed to characterize the functional roles of distinct KTI genes in *Populus* × *canescens*. Based on prior published evidence [41, 43], we selected three candidate genes – homologous to *Potri.019G124400*, *Potri.019G124600*, and *Potri.017G153200* – that exhibit differential responsiveness to herbivory. These genes were designated *KTI_400*, *KTI_600*, and KTI_53200, respectively, for ease of reference. In contrast to *KTI_400* and *KTI_600*, which occur in leaves*, *KTI_53200 was present in the xylem sap of *P.* x *canescens*, where it might play a role in poplar immune responses [43]. We validated the transcriptional responses of the candidate genes to wounding and phytohormone exposure. Subsequently, we generated gene knockout mutants using the CRISPR-Cas12a approach. We also produced constitutive overexpression lines under the *p35S* promoter to establish their role in herbivory defense and growth performance. We investigated the phenotypes of the transgenic poplar lines and performed herbivore feeding assays with *Helicoverpa armigera*.

## Methods

### Poplar propagation and growth conditions

We used hybrid poplar *Populus tremula* x *P. alba* (syn. *Populus* x *canescens,* INRA 717-1B4) for all experiments. We cloned poplar plantlets by microcuttings on half-strength MS medium [60] with vitamins (Duchefa Biochemie B.V., Haarlem, Netherlands) as reported previously [59]*.* The cuttings were grown under long day light conditions [16 h light, 70—85 µE m^−2^ s^−1^ PAR (photosynthetic active radiation), light source: L18W/840, Osram, Munich, Germany, 60% RH (relative air humidity) at 24 °C for approximately 4 weeks. We sliced leaves and stems of the plantlets for transformation experiments and used rooted plants for growth, phytohormone treatments and bioassays.

### Phylogenetic analysis and selection of candidate genes

For the phylogenetic analysis, putative KTI polypeptide sequences were searched in the *Populus trichocarpa* database PlantGenIE (https://plantgenie.org/; Date accessed: 28th August 2023) and in sPta717 v2 *P.* x *canescens* from AspenDB (https://www.aspendb.org/downloads; Date accessed: 28th August, 2023). Since *P.* x *canescens* (INRA 717-1B4) is a hybrid, gene model searches were performed for both parents, *Populus tremula* and *Populus alba.* Polypeptide sequences of these gene models were predicted by searching the Open Reading Frame (ORF) and their translation to amino acids. The prediction of ORFs and polypeptide translation, followed by multiple sequence alignment of the putative KTIs from *P. trichocarpa, P. tremula* and *P. alba,* were performed in Geneious Prime (Biomatters Ltd., Auckland, New Zealand; version: 2023.12) applying the Clustal Omega 1.2.2., mBed algorithm. The Geneious Tree Builder was used for the development of the phylogenetic tree with genetic distance model, Jukes-Cantor, tree build method, unweighted pair group method with arithmetic mean (UPGMA), bootstrap of 1000 replicates, and support threshold of 100% (Additional Figure S1).

In a previous study, we exposed *P.* x *canescens* WT to poplar leaf beetle (*Chrysomela populi*) in cages under outdoor conditions [41]. We downloaded the transcriptome of control and beetle-fed leaves of *P.* x *canescens* from this experiment [41] and searched the putative *KTI*s by their Potri.IDs (Additional Table S1). We extracted the mean transcript abundances of significantly differentially expressed *KTI* genes and clustered them with ClustVis [(http://biit.ut.ee/custvis/, accessed 20th May 2025, [56]]. We also searched the xylem sap of *P.* x *canescens* for the presence of KTIs [43]. Based on their expression profiles and presence or absence in xylem sap, we selected three candidate genes “Potri.019G124600”, “Potri.019G124400” and “Potri.017G153200”, hereafter called KTI_400, KTI_600 and KTI_53200, respectively.

### Prediction of signal peptides

The polypeptide sequences across the haplotypes of *P. tremula* and *P. alba* (Additional Figure S2) were used as a search query for the *in-silico* prediction of signal peptides in KTI_400, KTI_600 and KTI_53200. The databases SignalP-6.0 (https://services.healthtech.dtu.dk/services/SignalP-6.0/; accessed on 29th March, 2025) and PrediSI (http://www.predisi.de/; accessed on 29th March, 2025) were used for the predictions of the signal peptide. WoLF PSORT (https://www.genscript.com/wolf-psort.html; accessed on 29th March, 2025) and Plant-mSubP (https://bioinfo.usu.edu/Plant-mSubP/; accessed on 29th March, 2025) were used for the prediction of the sub-cellular localization.

### Transformation of poplar

For the transformation of poplar, we designed vectors, cloned them in *E. coli*, transformed them into *Agrobacteria*, which were then used to transform poplars, adapting protocols from [15] and [4]. The details of the adapted pipeline have been described in the Additional “Methods”. Briefly, the CRISPR-Cas12a gene knock-out and cloning strategies were adapted from Merker et al., [55]. The Gateway-compatible cloning plasmid sets, pDettLbCas12a and pEnRZ-Lb-Chimera were a gift from Prof. Dr. H. Puchta (KIT, Karlsruhe, Germany) and were used for poplar transformation. For the target site design, the PAM (Protospacer Adjacent Motif) site of the CRISPR-Cas12a system (5’-TTTV-3’) was initially searched within the exon regions of *KTI_400, KTI_600,* and KTI_53200*.* Subsequently, 24-nucleotide Target_400_600 and Target_53200 sites, lying at the 3’ site of the PAM were used for the double (*KTI_400* and *KTI_600*) and single (KTI_53200) knock-out sites. To ensure the specificity of the target sites, the genome of *P.* x *canescens* (version 2, *P.* x *canescens*, https://www.aspendb.org/downloads; Date accessed: 4th September 2023) was searched to exclude off-targets. We also generated empty vector control lines comprising only the ubiquitin (*ubq)* promoter.

Over-expression lines of the candidate *KTIs* were generated under the *p35S* promoter, using a binary vector set pDONR201 (entry vector; Invitrogen Life technologies) and pK7WG2 (destination vector; [42]), which are Gateway compatible. The CDSs of *KTI_400, KTI_600* and KTI_53200 were individually cloned into the plasmid vector sets (Additional Methods). We also produced empty vector lines containing only the *p35S* promoter in the destination vector pK7WG2.

For plant transformation, we used excised, slit leaves and stems from three-week old sterile poplar plantlets, which were co-cultivated with *Agrobacterium tumefaciens* (GV3101) containing the desired gene construct. Calli of the co-cultured tissues were induced on a callus-inducing medium containing gentamicin (60 mg L^−1^) for CRISPR-Cas12a or kanamycin (50 mg L^−1^) for the *p35S* transformed poplars in climatized cabinets (AR-75L, Percival Scientific) at 28 °C, 20 µE m^−2^ s^−1^ PAR, 60% RH, 16 h of light (light source: Alto 32 Watt, Philips, Amsterdam, Netherlands). The emerging shoots were transferred to a rooting medium containing either gentamicin (60 mg L^−1^) or kanamycin (50 mg L^−1^) and grown at 28 °C, 60 µE PAR with 16 h light for 4 weeks (light source: Alto 32 Watt and LG4507.4).

To control the insertion of T-DNA and for genotypic analyses of the mutant poplar lines, DNA was extracted from 100 mg of frozen, milled leaf tissues using the innuPREP Plant DNA kit (Analytik Jena GmbH, Jena, Germany) according to the manufacturer’s instructions and analysed by PCR (see Additional “Methods”, Primers for cloning and the PCRs are presented in Additional Table S2). The gene editing patterns introduced due to the CRISPR-Cas12a systems and the insertion of the overexpression constructs were confirmed via Sanger sequencing (Microsynth SeqLab, Göttingen, Germany) of the PCR amplicons, flanking the target sequence.

### Real Time Quantitative Polymerase Chain Reaction (RT qPCR)

Frozen leaves (100 mg) were milled to a homogeneous frozen powder (MM400, Retsch GmbH, Haan, Germany) under cooling to prevent thawing. We used the innuPREP Plant RNA kit (Analytik Jena GmbH) for RNA extraction according to the manufacturer´s instructions. The resulting RNA was eluted with RNase-free water in a volume of 30µL. Total RNA yield was measured spectrophotometrically using NanoDrop™ One spectrophotometer (Thermo Fisher Scientific, Wilmington, Delaware, US). One µg of RNA was used to synthesize cDNA with RevertAid First Strand cDNA Synthesis kit (Thermo Fisher Scientific), as described by the manufacturer’s protocol.

The transcript level quantification was performed using RT-qPCR with innuMIX qPCR DSGreen Standard (Analytik Jena GmbH), following the manufacturer’s guidelines with the following PCR conditions: initial denaturation at 95 °C for 2 min, denaturation at 95 °C for 10 s, annealing temperature specific to the primer (Additional Table S2 for annealing temperature of primer pair) for 10 s, elongation at 72 °C for 20 s (fluorescence read at this step), 45 cycles of denaturation to elongation. The transcript abundances were analyzed with the ddCt method (qSOFT program, version: 4.0, Analytik Jena GmbH) [63]. Two house-keeping genes *ACTIN* and *UBIQUITIN* were used for the normalization. All primer pairs were tested for their efficiency [63]. The primers are specified in Additional Table S2 and Additional Figure S3.

### Wounding experiments

Rooted WT poplars were potted in “N-type soil” (Hawita Gruppe GmbH, Vechta, Germany) and acclimated to greenhouse conditions (16 h light, 21 to 28 °C, 150 µE m^−2^ s^−1^ light conditions, approximately 30% relative air humidity, light source: 163 15L34, Adolf Schuch, Worms, Germany) as described by Müller et al. [59]. Plants were irrigated with tap water every alternate day and were randomized weekly. For foliar mechanical wounding experiments, the third fully developed leaf from the top of eight-week-old poplars was used. Wounding was performed with a micro-tissue tweezer, 1 × 2 teeth (Prestige 7–102) by punching 15 to 20 holes, distributed homogenously on the leaf. We chose sampling time points and leaf position similar to previous studies [65, 78]. After three and eight hours, the wounded leaf was excised and flash-frozen in liquid nitrogen. Control samples were the third fully developed unwounded leaf from the top of independent plants (n = 3 to 4 individual plants per treatment). To avoid bias by stress volatiles, wounded and control plants were separated in independent greenhouse cabinets for the experiments. The leaves were used to determine the transcript abundances of target genes as described above.

### Phytohormone treatments

For exogenous phytohormone application, ten-week-old greenhouse-grown WT poplars were divided into separate greenhouse cabinets and exposed to one of the following treatments: meJA (methyl jasmonic acid, 200 µM dissolved in demineralized water, Merck KGaA, Darmstadt, Germany), ACC (aminocyclopropane-1-carboxylic acid, 100 µM, dissolved in demineralized water; Merck KGaA), meJA- and ACC-mock solution (demineralized water), BTH (Benzothiadiazole 1000 µM, dissolved in 10% methanol; Merck KGaA) and BTH mock solution (10% methanol). All phytohormone and mock solutions were Additionaled with 0.1% Tween-20 (Merck KGaA) to improve adhesion to the leaves. The plants were individually sprayed on the leaves' abaxial and adaxial surfaces until drip-off. After spraying, plants were immediately enclosed in a polypropylene bag (400 × 780 mm, Labsolute, Th. Geyer GmbH and Co. KG., Höxter, Germany) for 4 h. Subsequently, the plants were grown for an additional 4 h and 20 h without polypropylene bags before tissue sampling. This resulted in sampling time points of 8 h and 24 h. The first fully developed leaf from the top was harvested, shock-frozen in liquid nitrogen and used to determine the transcript abundances of target genes. Each treatment was conducted with *n* = 3 to 4 individual plants.

### Physiological and phenotypic characterization of transgenic poplars

Rooted plants of the WT, the CRISPR-Cas12a knock-out lines (= *kti* lines), the *p35S KTI* overexpressing lines (*KTIox* lines) and empty vector control lines for *KTIox* and *kti* were potted and acclimated to greenhouse conditions (20–28 °C, 150 µE m^−2^ s^−1^ PAR, 16 h light achieved by ambient light in addition to irradiation with LED lamps 163 15L34, Adolf Schuch, Worms, Germany; approximately 30% relative air humidity). The plants were daily irrigated with tap water and grown for two months. Gas exchange was determined four times in bi-weekly intervals on the third fully expanded leaf with a portable photosynthesis system device (LCpro +, ADC BioScientific Ltd, UK) under ambient light conditions. At harvest, leaves, stems and roots of all plants were weighed, dried (2 weeks at 60 °C) and used to determine whole plant biomass. We used 2 or 3 independent transgenic lines per transformation event (empty vector controls, the *KTIox* and the *kti* lines), each with four plants and the WT (n = 8).

### Bioassay of transgenic poplars with *Helicoverpa armigera*

The eggs of the broad-range generalist insect *Helicoverpa armigera* (provided by Prof. Dr. M. Rostás, Agricultural Entomology, Department for Crop Sciences, University of Göttingen) were allowed to hatch in a plastic container (8 cm length × 13.5 cm width × 6 cm height) at 22 °C and 16 h light [60 μE m^−2^ s^−1^ PAR (provided by Alto 32 Watt, Philips, Amsterdam, Netherlands)], 60% RH in a climate cabinet (AR-75L, Percival Scientific, Perry, USA), containing an artificial diet composed of alfalfa powder, rapeseed oil, baker’s yeast, Wesson Salt Mix, β-sistosterol, L-leucine, ascorbic acid, vitamin mix, sorbic acid, bean flour and 4-hydroxybenzene S [33]. The larvae were used when they reached their 1st to 2nd instar stage (determined after [57] and had lengths of approximately 1 to 3 mm. The starting weight was determined for pools of 10 larvae on an analytical balance (Cubis® MCA225S-2S00-I, Sartorius, Göttingen, Germany) and divided by 10.

Two- to 3-week-old rooted WT and transgenic poplar plants from stock cultures were individually placed on solid ½ MS media in sterile squared Magenta jars (size: 76 × 76 × 102 mm Magenta, Merck KGaA). The plants were cultivated for one week in climatized cabinets (Percival Scientific, Perry, USA; 22 °C, 60 μE m^−2^ s^−1^ PAR, 16 h light, light source: Alto 32 Watt). A single *H. armigera* larvae was placed in each Magenta jar containing one poplar plantlet. After 12 days of feeding, the weight of each individual *H. armigera* was measured on the analytical balance (Cubis® MCA225S-2S00-I, Sartorius). Weight gain was determined as (Weight after feeding—Weight at the beginning). Two independent experiments were conducted, each included WT, empty vector controls, and 2 lines per *kti* and *KTIox*. The number of individual plants per experiment varied and is indicated in the figure legend.

### Statistical analyses

All statistical analyses were performed in R (R Core Team, 2022) and were visualized in RStudio (RStudio Team, 2020) or using OriginPro2024b (Northampton, Massachusetts, USA). Data were checked for normal distribution and variance homogeneity (Levene´s test, visual inspection of residuals). ANOVA was performed using general linear models with the package "multcomp" [38] followed by a post-hoc test (usually Tukey). When the data were not normal-distributed, we used the Kruskal–Wallis test for pairwise comparisons. When more than one experiment was analyzed, packages "car" [32] and "lme4" [8] were used to assign random effects to every experiment. Differences between means of treatments and controls were considered significant at *p* < 0.05.

## Results

### In-silico selection of potential candidate KTIs

We conducted multiple sequence alignment of the amino acid sequences inferred from the *P.* x *canescens* genome (i.e., the parent´s genomes *P. alba* and *P. tremula*) together with the *P. trichocarpa* genome and identified a total of 28 and 29 *KTI* sequences in the haploid parent genomes of *P.* x *canescens* (Additional Fig. S1). In this study, we used the Potri IDs of the closest *P.* x *canescens* homologs for annotation to ease the comparison among studies. The Potra IDs are shown in Additional Table S4.

The putative KTIs clustered in three large clades with 8 to 10 members (Additional Fig. S1). Two KTIs (Potri.019G088200 for both parents and Potri.003G097900 only for *P. alba*) did not cluster with any of the larger groups (Additional Fig. S1).

**Fig. 1:**
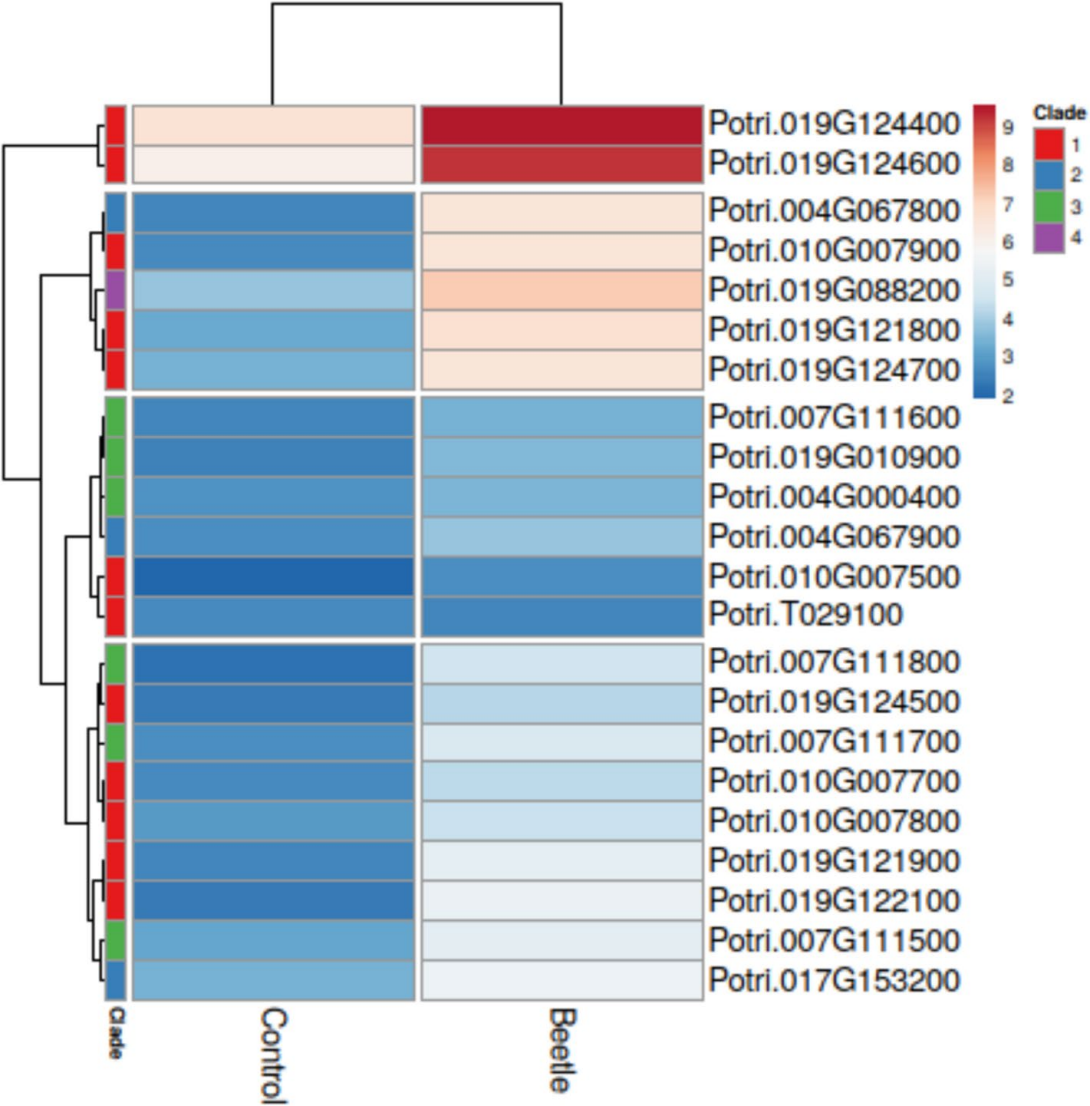
Differentially expressed *KTI* genes (*p*_*adj*_ < 0.05) after exposure of *P*. x *canescens* to *C. populi* in outdoor cage areas. Data were extracted from the Additional material of RNAseq results in Kaling et al. [41]. Means were clustered in rows. Left panel “clade” indicates the phylogenetic clade of the genes

To identify poplar KTIs responsive to herbivory, we analyzed a transcriptomic dataset from *Populus* × *canescens* exposed to *Chrysomela populi* [41] and extracted the expressed *KTI*s. Twenty-two of the *KTI*s in *P.* x *canescens* showed significant upregulation of transcript abundances in response to poplar leaf beetle feeding (Fig. 1). Genes with the greatest increases in transcript abundances in response to herbivory were KTI_400 and KTI_600 (both in clade I of the phylogeny, orthologue to TI3 in *P. trichocarpa* x *deltoides*, [49]). Further relatively strong transcriptional responses were observed for a group of five genes, including members of clade I and clade II in addition to the single *KTI,* Potri.019G088200 (Fig. 1). *KTI,* Potri.019G088200 is an orthologue to *P. nigra PnD1* [28]. The remaining genes showed moderate or low transcriptional regulation and comprised members of clade I, II, and III of the phylogeny (Fig. 1).

For further analyses, we selected *KTI_400* and *KTI_600* (clade I) because of their massive response to herbivory. Furthermore, KTI_53200 (clade II, orthologue to *KTI5* in *P. alba* [13]*,*) with a moderate herbivory response (Fig. 1) was chosen.

### Molecular features of potential candidate KTIs

Among the selected candidates, KTI_400 and KTI_600 exhibited 92% identity of the amino acid sequences, and both had approximately 70% identity with their closest neighbors Potri.019G121900, Potri.019G124500, Potri.019G124700, and Potri.019G122100 (Additional Fig. S1, Fig. S2). With the exception of Potri.019G124700, the close neighbors showed only very low responsiveness to herbivory (Fig. 1). Candidate KTI_53200 shared 67% amino acid sequence identity with its closest neighbour Potri.004G067900, a gene also showing only low induction by herbivory (Fig. 1). Owing to a relatively large dissimilarity within the amino acid sequences, KTI_53200 shared only 19% identity with KTI_400 and 36% with KTI_600 (Additional Fig. S2). KTI_400 and KTI_600 showed 47% amino acid identity with their closest *Arabidopsis thaliana* orthologue At*KTI3* (At1g73325) and KTI_53200 51% with At*KTI5* (At1g17860).

**Fig. 2:**
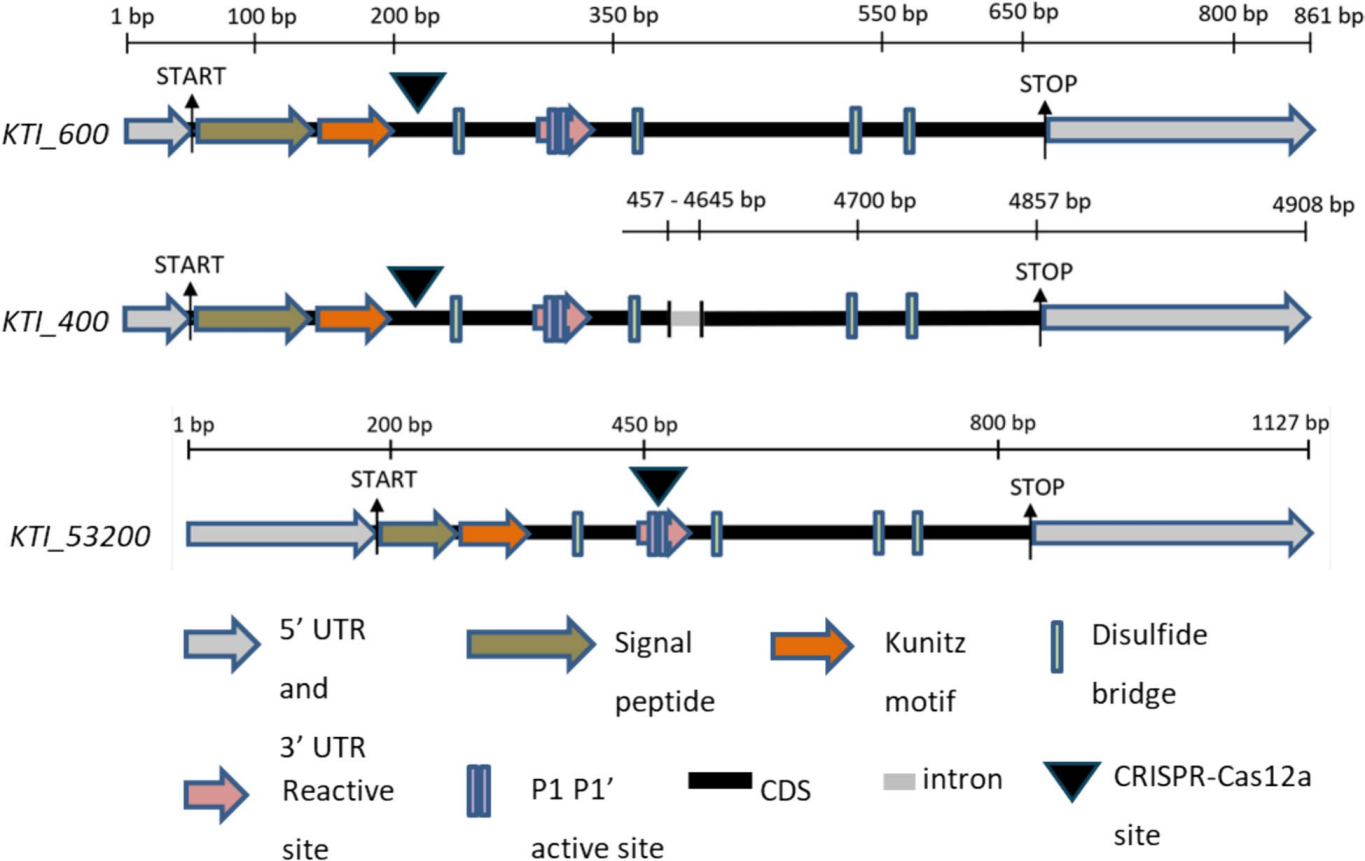
Gene models of candidate *KTI_400*, *KTI_600* and KTI_53200. Motifs of Kunitz Trypsin Inhibitor marked with colored arrows and boxes. The length of genomic DNA is stated above the respective sequence. The cDNA/amino acid lengths are *KTI_400:* 932 bp/203 aa, *KTI_600:* 861 bp/203aa and KTI_53200*:* 1122 bp/210aa. Start and stop codons are marked with black arrows. All sequences have been retrieved from sPta717 v2 (www.aspendb.org/databases; date accessed: 21.^st^ August, 2020)

Each of the three candidate KTIs, KTI_400, KTI_600 and KTI_53200 was characterized by a signal peptide (Fig. 2, Additional Fig. S2 for the amino acid sequence), which predicted extracellular localization with high probability (Additional Table S5). However, a note of caution is warranted because no consistent prediction for subcellular localization was observed when other prediction programs were used (Additional Table S5). Furthermore, the candidate KTIs contained the characteristic Kunitz motif ([L, I, V, M]-X-D-X2-G-X2-[L, I, V, M]-X5-Y-X-[L, I, V, M]) and six cysteine residues, of which four are predicted to form disulfide bridges (Fig. 2, Additional Fig. S2). The P1-P1’ motif in the reactive sites, which interact with the proteases, were “E-S” (glutamic acid-serine) residues at the 86–87 aa position of KTI_400 and KTI_600, and “D-D” (aspartic acid-aspartic acid) residues at the 89 aa position of KTI_53200 (Additional Fig. S2).

### *In-planta* response to wounding and phytohormone treatments of the candidate *KTI*s

**Fig. 3:**
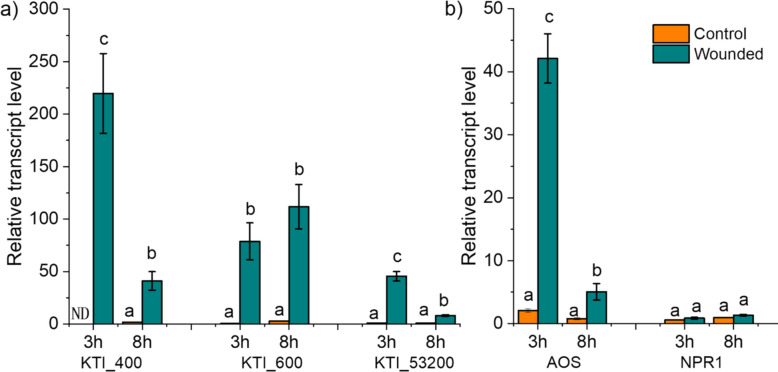
Relative transcript abundances of (**a**) candidate *Kunitz Trypsin Inhibitor* (*KTI_400, KTI_600,* and KTI_53200*)* and (**b**) jasmonic acid and salicylic acid marker genes (*AOS* and *NPR1*) in response to wounding of *Populus* x *canescens* leaves. Relative transcript abundances were determined at 3 and 8 h post wounding. The third leaf from the apex of 8-week-old poplars grown in soil in the greenhouse were used for wounding. Transcript levels were tested via RT-qPCR. The third leaf from non-wounded plants, sampled at the same time points, were used as controls. Transcript abundances of the target genes were expressed relative to the reference genes *ACTIN* and *UBIQUITIN*. Bars show means of n = 3 to 4 ± SD biological replicates. Each biological replicate is from an independent plant. Different letters above bars per gene represent significant differences at *p* < 0.05 (ANOVA, Tukey post-hoc test). ND, non-detectable

The candidate *KTI*s showed significant increases in transcript abundances 3 h and 8 h after mechanical wounding of leaves (Fig. 3a). The initial wounding response was greatest for *KTI_400* with up to 200-fold increases in transcript levels, but declined afterward, whereas *KTI_600* was less induced (approximately 40-fold) but remained stably increased after 8 h (Fig. 3a). KTI_53200 showed less responsiveness to wounding than *KTI_400* and *KTI_600* (Fig. 3a).

Since wounding responses may imply regulation by phytohormones [29], we studied marker genes for the JA and SA pathways, *ALLENE OXIDE SYNTHASE* (*AOS*) and *NON-EXPRESSOR OF PATHOGENESIS-RELATED GENES 1* (*NPR1*), respectively. We observed divergent responses of *AOS* and *NPR1* to wounding (Fig. 3b). *AOS* transcript abundances were increased after 3 h and 8 h, similar to the pattern observed for *KTI_400* and KTI_53200, whereas *NPR1* was unaffected (Fig. 3b).

**Fig. 4:**
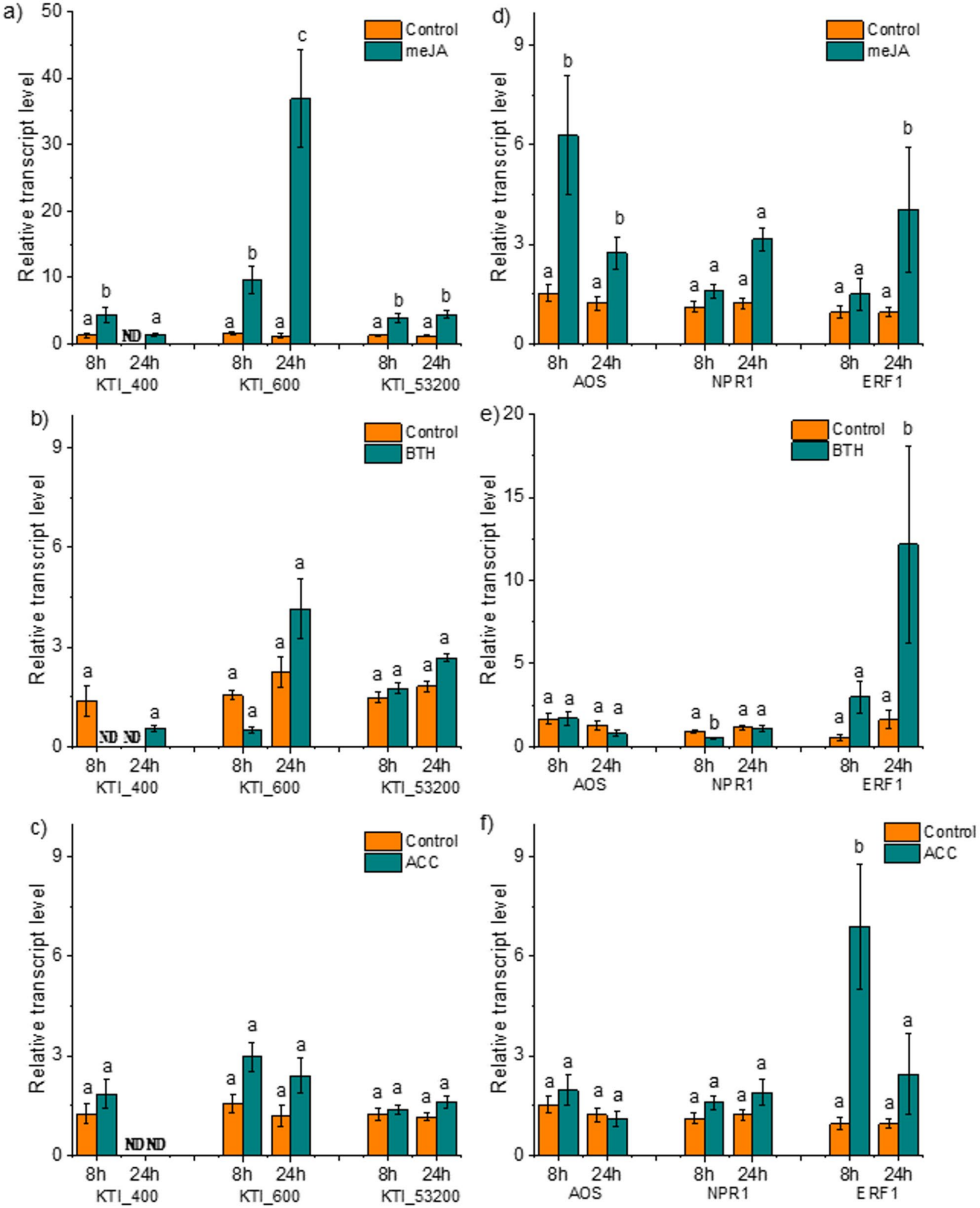
Response of the candidate *Kunitz Trypsin Inhibitors*, jasmonic acid, salicylic acid and ethylene marker genes 8 h and 24 h after exogenous phytohormone spraying. **a**,**d** meJA (200 uM); **b**,**e** BTH (1000 µM); c,f) ACC (100 µM) were sprayed to *Populus* x *canescens*. Bars represent the mean transcript abundance of *KTI_400, KTI_600, *KTI_53200*, AOS,* *NPR1* and *ERF1* normalized to the reference genes *ACTIN, UBIQUITIN,* and mock-treated controls, *n* = 3 or 4 (biological replicates). Plants were separated in different chambers, and meJA, BTH, and mock solution were sprayed on the leaves until dripping. Transcript abundance was quantified via RT-qPCR. Error bar represents the standard deviation. Different letters above bars per gene represent significant differences at *p* < 0.05 (ANOVA, Tukey post-hoc test) between control and treatment. ND, non-detectable. meJA, methyl-jasmonic acid; BTH, Benzothiadiazole; ACC, 1-Aminocyclopropane-1-carboxylic acid

To obtain further evidence for phytohormone responsiveness of the candidate KTIs, we treated *P*. x *canescens* exogenously with meJA (JAs), BTH (SA analogue), or ACC (ethylene precursor). *KTI_400* showed significantly increased transcript levels (fivefold) at 8 h post meJA treatment and declined to the levels of the mock-treated controls at 24 h (Fig. 4a). The highest up-regulation was noted for *KTI_600* with approximately tenfold and 35-fold increases in expression levels at 8 h and 24 h post meJA treatment. KTI_53200 showed moderate increases in transcript abundances in response to meJA treatments (Fig. 4a). BTH and ACC exposure of poplar did not induce increased transcript levels of the candidate *KTIs* (Fig. 4b,c)*.*

Initially, we also included *KTI_Potri.019G088200* in our study. Unlike the candidate KTIs, this gene did not exhibit a rapid transcriptional response to wounding (Additional Fig. S4a). However, it showed a significant increase in transcript levels following meJA treatment, detectable at 8 h, while no induction was observed in response to ACC or BTH (Additional Fig. S4b).We further tested marker gene expression for the exogenously applied phytohormones to validate their *in planta* activity (Fig. 4d-f). *AOS* transcript levels were responsive to meJA at both tested time points, showing approximately 6- and twofold increases after 8 h and 24 h, respectively (Fig. 4d). For *NPR1,* a significant decrease at 8 h post BTH exposure was observed, whereas *AOS* was unresponsive to BTH and ACC treatments (Fig. 4e). *ETHYLENE RESPONSIVE FACTOR 1* (*ERF1*) transcript abundance increased in response to ACC (Fig. 4f) but also in response to meJA and BTH treatments (Fig. 4d,e).

### Phenotypes of poplar overexpression and knockout *KTI* lines

For poplar transformation, we chose a double knockout strategy, simultaneously targeting *KTI_400* and *KTI_600* (assigned as *kti4* + *600*) and a single knockout strategy for KTI_53200 (kti_53200)*,* using CRISPR-Cas12a. Positive CRISPR-Cas12a transgenic lines were tested by PCR, followed by Sanger sequencing of the CRISPR-Cas12a target region. The CRISPR-Cas12a construct targeting *KTI4* + *600* showed gene-editing patterns of 50, 14, 11, 10, 9, 8, 6 and 2 bp deletions, whereas the CRISPR-Cas12a construct targeting KTI_53200*,* showed deletions of 10, 8, 7, 6, and 5 bp (relevant mutant lines in Additional Table S6). The CRISPR-Cas12a mutant lines *kti4* + *600_1_28*, *kti4* + *600_1_52* and *kti53200_2_18*, and *kti53200_2_29* showed prominent frame-shift mutations and putatively premature stop codons (Additional Table S7).

Overexpression lines (*KTIox*) were generated by expression of each *KTI* individually under the *p35S* promoter. The transcript levels of the target candidate *KTI*s were quantified in 26 *KTIox* lines (four *KTIox_400* lines, eight *KTIox_600* and fourteen *KTIox_53200*, Additional Fig. S5). Compared with WT levels, elevated *KTI* expression levels ranged from 10- to 60-fold in most of the tested *KTIox_400* and *KTIox_600* lines (Additional Fig. S5a,b). The *KTIox_53200* lines showed very high overexpression levels ranging from approximately 500 to 12000 above the WT levels (Additional Figure S5c).

**Fig. 5:**
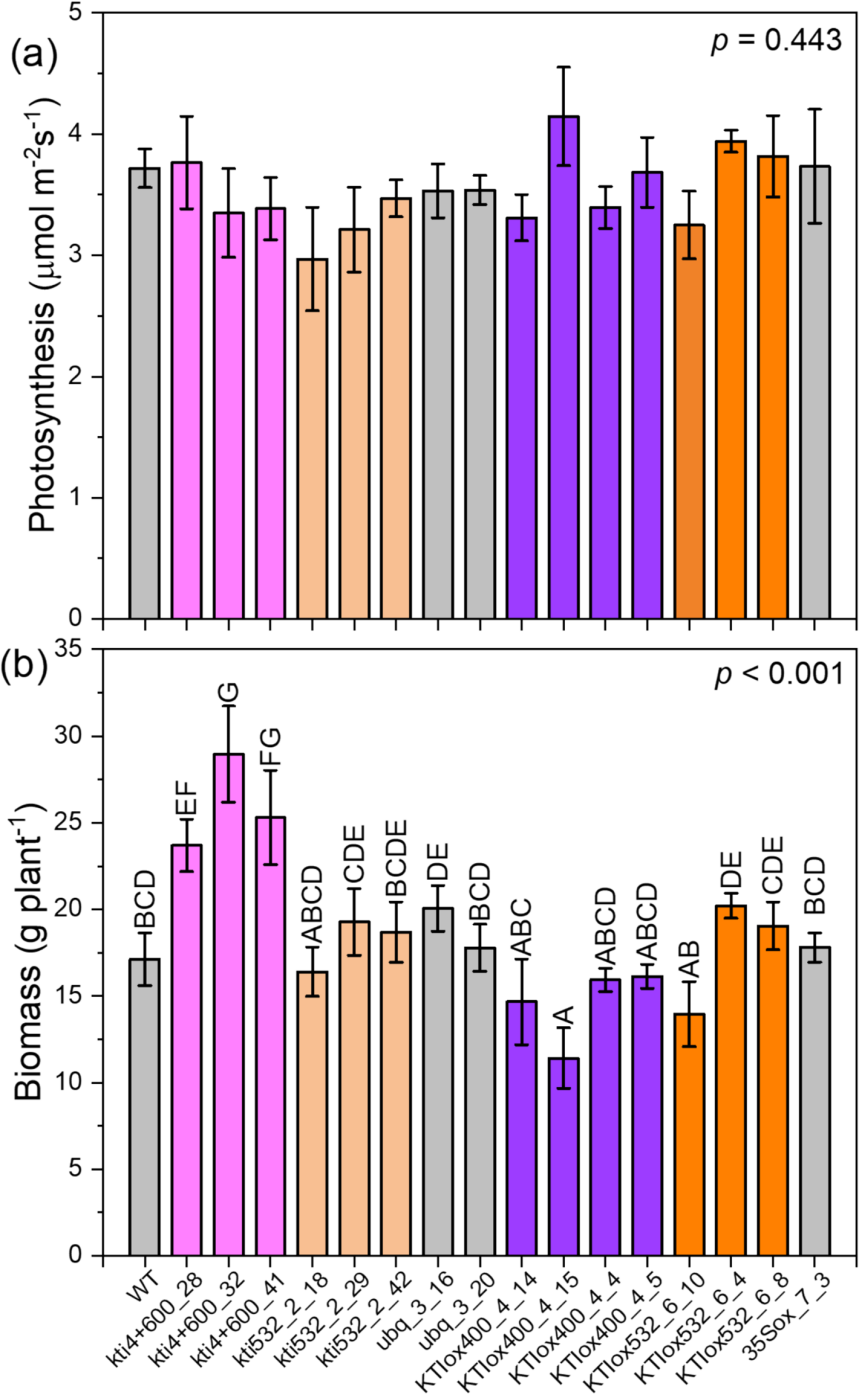
Photosynthesis (**a**) and biomass (**b**) of poplar WT and transgenic lines. The plants were grown for 2 months in greenhouse cabinets. *n* = 4 plants per line, WT: *n* = 8. Data show means ± SE. Inside the panels, p values for one-way ANOVA are shown. Different letters indicate significant differences of means at *p* < 0.05 (Tukey test)

We cultivated WT, empty vector lines, and *KTIox*, and *kti* lines under greenhouse conditions (Additional Fig. S6). There was no obvious visual phenotype (Additional Fig. S6). We did not observe significant differences in photosynthesis (Fig. 5a). Other physiological parameters (stomatal conductance, transpiration) and morphological parameters (plant height, stem diameter, biomass of leaves, stem, and roots, root-to-shoot ratio) either showed no or small but significant variations among the lines (Additional Table S8). However, the whole-plant biomass (sum of leaf, stem, and root) of the *kti4* + *600* lines was significantly greater than that of the other lines (Fig. 5b).

### Differences in herbivory on *KTI* overexpressing and knock out poplar lines

Initial attempts to grow the generalist *H. armigera* on *P.* x *canescens* leaves of greenhouse-cultivated plants as the sole diet were not successful due to high mortality of the larvae, whereas larvae on an artificial diet showed massive weight gain from approximately 0.4 mg at the start to approximately 19 mg within 12d. After testing various conditions, we found that exposing sterile-grown, “naive” poplar plantlets individually in Magenta jars to *H. armigera* resulted in reproducible results. Significant damage by herbivory was visually noted for all lines (Additional Fig. S7).

**Fig. 6:**
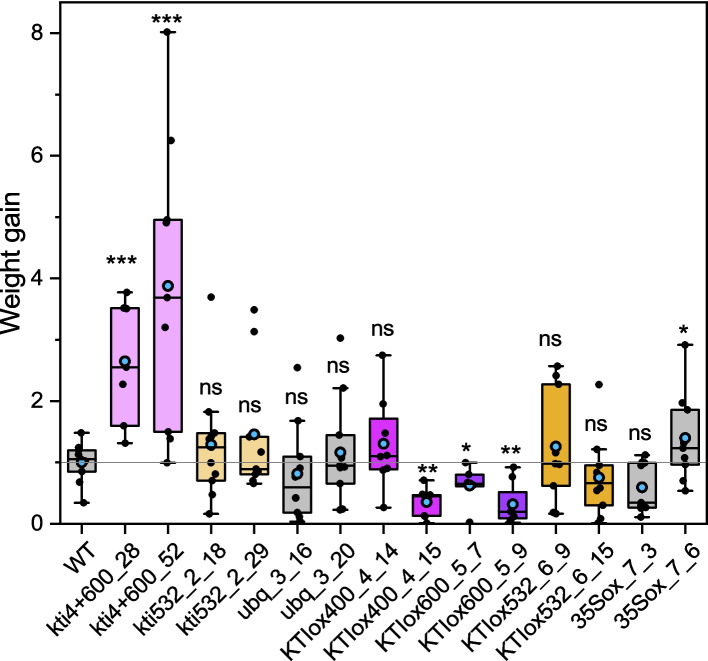
Weight gain of *Helicoverpa armigera* larvae on *Kunitz Trypsin Inhibitor* transgenic poplars. One II-instar stage larva was applied per plant to CRISPR-Cas12a loss-of-function *kti4* + *600_1_28*, *kti4* + *600_1_52*, *kti53200_2_18*, *kti53200_2_29*, empty control vector *ubq*::*3_16* and *ubq*::*3_20* lines; *35S* over-expressing *KTI400_4_14*, *KTI400_4_15*, *KTI600_5_7*, *KTI600_5_9, KTI53200_6_9* and *KTI53200_6_15,* empty vector control *35S::7_3* and *35S::7_6* and WT (Wild-type) growing individually in sterile jars. Six to 12 biological replicates per line from two separate experiments were used. The sterile jars containing one poplar plantlet and one larva were placed in growth cabinets for 12 days. In each experiment, the data were normalized to the mean weight gain of the wildtype. Individual data = black circles, horizontal line = median, blue circle = mean. Box extends from the 25th to 75th percentile, and whiskers encompass the minimum and maximum values. Statistical analysis was performed with ANOVA and Kruskal–Wallis post hoc test. Stars indicate significant differences compared to the controls: * *p* < 0.05, ** *p* < 0.01, ns = not significant

*H. armigera* larvae revealed significantly higher weight gain after feeding on *kti4* + *600_1_28* and *kti4* + *600_1_52* than on WT or on empty vector plants *ubq::3_16,* and *ubq::3_20* (Fig. 6). The weight gain of the larvae feeding on these mutant lines was approximately three-fold higher than on WT plants. In contrast to the *kti4* + *600* poplars, there were no significant differences among kti_53200 lines to the WT or empty vector lines (Fig. 6).

In the *p35S-*overexpression lines, we observed about 40% less weight gain for larvae feeding on *KTIox_600_5_7* and about 70% less weight gain for larvae feeding on *KTIox_400_4_15* and *KTIox_600_5_9* but no difference to the WT for *KTIox400_4_14* (Fig. 6)*.* No significant weight differences were observed for *H. armigera* larvae feeding on *KTIox_53200* lines compared with the WT or empty vector line *35Sox_7_3*, while the weight gain on empty vector line *35Sox_7_6* was slightly but significantly increased compared with the WT (Fig. 6).

## Discussion

### Phylogeny and expression patterns of poplar *KTI*s

In this study, we determined the phylogeny of KTIs in *P*. x *canescens* based on full-length amino sequences, used an *in-sili*co strategy to identify *KTI*s responsive to herbivore feeding, tested the selected genes for their induction by wounding and phytohormones, and used them to dissect their functions as herbivore protectants by forward and reverse genetic engineering. We identified 28 *KTI*s in the haploid genome of *P.* x *canescens,* which compares well with the number of KTI genes reported for other *Populus* species or hybrids (31 KTIs in *P. trichocarpa* x *deltoides* [65], 31 KTIs in *P. trichocarpa* [48], 32 KTIs in *P. nigra* [28]*,* and 29 KTIs in *P. yunnanensis* [35]) and underpins massive expansion of this gene family compared with other species (e.g., seven KTIs in the herbaceous model plant *Arabidopsis thaliana* [5], three KTIs in *Camellia sinensis* [87], and four in *Vitis vinifera* [35]). In line with previous assessments [9], we observed the typical structural characteristics for the KTIs (signal peptide, P1-P1´site, cysteine residues, Kunitz motif) in *P.* x *canescens* and confirmed that the KTIs clustered in three major clades and a small group IV, with only one member [28, 35, 48].

Several studies suggested that poplar *KTI* genes in clade I are involved in herbivory defense [22, 28, 49], that clade II is mixed, containing KTIs for defense and development (female catkins, apical shoot, flowers [19, 48, 49]), whereas functions of KTIs in clade III remain enigmatic. Using *in-silico* data from our previous study [41], we observed transcriptional responses to *C. populi* feeding for *KTI*s in all clades; however, distinct members in clade I showed stronger transcriptional induction than those from clades II and III. *KTI_8820* (clade IV) also showed notable upregulation under herbivory by *C. populi* (our study) and other lepidopterans [28] but we observed less massive meJA and wounding responses than for *KTI_600*. Whether genes with low basal transcript levels and low induction found in our study for many clade II and III genes contribute markedly to poplar defense is questionable since the production of KTI proteins is transcriptionally regulated [48]. Accordingly, increased *KTI* expression levels correlate with higher *in-vitro* trypsin inhibitor activity [28].

Phytohormones, especially JAs, are important for sensing and inducing defenses against herbivores [3, 29]. The formation of JAs is induced by elicitors and triggered by wounding, starting with lipid oxidation by lipoxygenases (LOX) [31]. Oxidized lipid precursors are then transformed by AOS into the unstable intermediate 12,13-epoxyoctadecatrienoic acid, which marks the entry into the JAs biosynthetic pathway [31]. In poplar leaves damaged by *C. populi*, increased levels of octadecatrienoic acid were observed, implying enhanced JA signaling [78]. In line with previous studies in other poplar species [18, 36, 49, 51, 65], we observed that wounding and meJA caused increased transcript levels of *KTI*s. The response intensities and time courses of the selected *KTI*s varied in our study, wounding stimulating *KTI*s in the order *KTI_400* > *KTI_600* > KTI_53200*,* whereas *KTI_600* showed greater induction by meJA than *KTI_400* and KTI_53200*.* In parallel, under both conditions of wounding or meJA exposure, *AOS*, a key enzyme for JA biosynthesis, was also induced, whereas BTH or ACC exposure did neither influence *AOS* nor our candidates´ *KTI* expression. At first glance, the induction of *AOS* by meJA is surprising as AOS is a key enzyme in JA biosynthesis [30]. Often, end-products of biosynthetic pathways exert feedback inhibition to regulate their own accumulation [23]. Since meJA is an end-product of the JA pathway, one might expect it to suppress further JA biosynthesis. However, in contrast to other regulatory pathways, JAs can enhance their own production through a positive feedback mechanism [72]. This has been demonstrated in *Arabidopsis thaliana* [7], *Camellia sinensis* (tea) [76], and *Taxus chinensis* (Chinese yew) [44]. The observed upregulation of *AOS* transcripts in poplars following exogenous meJA application suggests that JA biosynthesis may also be stimulated in this species under such conditions.

In contrast to meJA, exogenous ACC or BTH did not trigger KTI expression, although marker gene analyses suggested intracellular responses to these compounds. *ETR1*, a marker for ethylene [27], was upregulated after ACC (precursor for ethylene) application. The responses to BTH (as an SA analogue) were complex. A central function of SA is the activation of defenses against biotrophic pathogens via the receptor NPR1 [84]. We observed a twofold decrease in *NPR1* upon BTH treatment. Similarly, Ullah et al. [83] found only moderate variations in *NPR1* upon exposure to rust (*Melampsora larici-populina),* despite massive SA accumulation, presumably because inactive forms of NPR1 present in the cytosol are post-translationally activated by SA [46, 86]. Whether the increase in *ERF1* observed in our study under high BTH reflects the well-known antagonistic regulation of the SA and ethylene signaling pathways [79] is speculative, especially since these pathways are independently regulated in poplar [82]. Importantly, the application of BTH and ACC did not cause up-regulation of the transcript levels of any candidate KTI gene, which supports that SA and ET pathways were not involved in KTI induction. Hence, our results underpin a distinctive role of JAs for defense activation, likely downstream of JA production since the stimulation of *KTI*s was achieved in undamaged plants. In future studies, it will be interesting to dissect the molecular mechanisms resulting in divergent responses of distinct KTIs.

### Functional analysis of KTIs and ecological aspects

In previous studies, the functions of poplar KTIs were investigated in vitro or ectopically and revealed diversified patterns. For example, biochemical analyses of five recombinant KTIs from poplar showed different inhibitory profiles for commercial proteases as well as for proteases in midgut extracts from forest tent caterpillar (*Malacosoma disstria*) and armyworm (*Mamestra configurata*) [49]. However, the greatest activity in the biochemical assays was exerted by a soybean (*Glycine max*) protease inhibitor (GmKTI) [49]. Overexpression of *GmKTI* in poplar (*P. nigra* resulted in inhibitory activity in transgenic leaf extracts; yet this transformation did not affect larval growth of polyphagous lepidopterans, *Lymantria dispar* and *Clostera anastomosis* feeding on the transgenic plants [21]*.* Ectopic overexpression of poplar KTIs (*PtdKTI2*, *PtdPOP3*, *PtdWIN4*, *PtKTI5*) in *Arabidopsis* resulted only in moderate reductions of larval growth but hampered proper larval development [39]. In contrast to those preceding studies, we used *P.* x *canescens* as the gene donor and host, employing overexpression under the constitutive *pS35* promoter and CRISPR-Cas12a for gene editing. The CRISPR-Cas12a system for plants was developed in *Arabidopsis thaliana*, where 21% of editing efficiency was noted [74]. We obtained similar editing efficiencies. The deletions ranged from 2 to 50 bp and occurred at the 3’ distal side of the PAM as observed in rice and *Arabidopsis thaliana* [10, 52, 74].

Using this novel approach, we clearly showed that KTI_400 and KTI_600 are central in regulating the fitness of a generalist herbivore. Increased expression levels similar to those induced by leaf wounding or meJA exposure caused significant reductions in the weight gain of *H. armigera*, whereas *kti4* + *600* lines made leaves more palatable for the larvae. The greatest weight gain compared to controls occurred when the larvae fed on CRISPR-Cas12a mutant lines with the largest deletions. We did not attempt to generate single knock-out lines of *KTI_400* and *KTI_600* since their responses to meJA, wounding, and *C. populi* feeding were similar, suggesting gene redundancy [24]. This presumption has yet to be tested. In *Arabidopsis thaliana*, suppression of single *KTI*s (*AtKTI4* and *AtKTI5* T-DNA insertion lines) effectively increased fecundity of spider mites (*Tetranychus urticae*) feeding on the transgenic plants [5]. However, the *Arabidopsis thaliana KTI* family is relatively small. Therefore, redundancy effects may be more likely in species with expanded *KTI* families such as *Populus*, especially for genes that share high similarity such as *KTI_400* and *KTI_600*. In our study, *KTI_400* and *KTI_600* shared the greatest similarities with four genes (Potri19G121900, Potri19G122100, Potri19G124500, and Potri19G124700), which did not show strong transcriptional responses to herbivory in the WT. Still, we cannot exclude that enhanced expression of these genes may partially compensate for KTI suppression in the kti4 + 600 lines. Thus, future studies (e.g., RNAseq, metabolomic and proteomic experiments) are necessary to characterize the specificity of *KTI_400 and KTI_600* suppression against a wider range of herbivores and their impact on plant metabolism.

In contrast to *KTI_400* and *KTI_600*, neither overexpression of KTI_53200 nor its suppression in CRISPR-Cas12a lines affected the growth of the leaf-feeding *H. armigera* larvae. Previous transcriptomic studies of poplar tissues, e.g., *P. trichocarp*a [77], found the highest expression levels of KTI_53200 in roots [35]. Since the KTI_53200 protein is abundant in the xylem sap of *P.* x *canescens* [43], it is possible that KTI_53200 is produced in roots and transported upward the stem together with a wealth of other proteins [25, 66]. A possible function of KTI_53200 could be the control of serine proteases, which are also present in poplar sap [43]. However, this idea is speculative and needs further studies. Other possibilities are functions in plant development [11, 37], abiotic stress protection [40], pathogen defense [16], or herbivore specificity [28].

Here, we tested only the generalist herbivore, *H. armigera*. *H. armigera* is a devastating pest in the (sub-)tropics, especially in Africa and Asia [45] but is also spreading across Europe and America (https://gd.eppo.int/taxon/HELIAR/datasheet). So far, its invasion into northern latitudes was limited by low winter temperatures, which prevented its survival. However, with increasing global temperatures, it is expected that *H. armigera* will become a threat to plants at northern latitudes [81]. Our study demonstrates the importance of KTI_400 and KTI_600 as defense against this pest under controlled conditions. In future studies, it will also be necessary to test the interaction of genetically modified KTI poplars with herbivores under field conditions, in particular with respect to their impact on the fitness of specialist poplar pests such as *C. populi* and *Phratora vitellinae* [14, 69].

An unexpected result of our study was that the *kti4* + *600* lines showed greater biomass production than overexpressing or control poplars. This suggests that KTI production imposes a fitness cost, potentially at the expense of growth. In a previous study, fitness costs associated with KTI in *Nicotiana attenuata* were only observed when plants with varying KTI levels were grown in close proximity, with lower-KTI plants producing more seeds, indicating a competitive disadvantage under belowground resource competition for plants with high KTI levels [85]. While this supports the classical view that defense and growth are trade-offs driven by resource allocation, alternative explanations beyond simple optimization of resource use efficiency are possible. Recent advances in ecological theory have reinterpreted growth-defense trade-offs in light of molecular regulation, emphasizing the role of phytohormones and signaling networks in coordinating these processes [58]. These insights suggest that growth and defense may not always be tightly coupled, opening new avenues for understanding the mechanistic basis of plant resource allocation. The poplars generated in our study will be an ideal tool to dig deeper into the molecular mechanisms linking intrinsic KTI production with plant and insect fitness traits. Further investigation into the specific mechanisms and efficacy of KTIs in poplar defense systems could pave the way for developing novel biocontrol strategies against herbivorous pests.

## Conclusions

*KTIs* are differentially regulated in response to feeding of the poplar specialist *C. populi*. Overexpression of the *KTI*s with the strongest transcriptional response had negative effects on the fitness of larvae of the generalist *H. armigera*, while knockout mutants showed increased larval growth. These results indicate a protective potential of KTI_400 and KTI_600 against the herbivore *H. armigera* and open perspectives for novel biocontrol measures.

## Supplementary Information

Additional file 1: Additional Figure S1: Phylogenetic tree of putative Kunitz Trypsin Inhibitors in Populus trichocarpa and the haplotype sequences of P. tremula and P. alba. Additional Figure S2: Multiple sequence alignment of amino acids of the three candidate Kunitz Trypsin Inhibitor proteins. Additional Figure S3: Linear curves for primer efficiencies. Additional Figure S4: a) KTI transcript levels of P. x canescens plantlets grown and wounded under sterile conditions, b) KTI (Potri019G08220, KTI_8220) transcript levels of greenhouse grown poplars after exposure to methyl-jasmonate (meJA), ACC or BTH. Additional Figure S5: Transcript abundances of KTI_400, KTI_600 and KTI_53200 over-expressed under the 35S promoter. Additional Figure S6: Twelve-week-old Kunitz Trypsin Inhibitor mutant lines grown under greenhouse conditions. Additional Figure S7: Representative photographs of Kunitz Trypsin Inhibitor poplar mutant lines under constant feeding of Helicoverpa armigera larvae. Additional Table S1: Data set for potential KTIs extracted from Kaling et al. 2018. Additional Table S2: List of all primer sets used for cloning and standard PCR. Additional Table S3: List of primers for RT-qPCR. Additional Table S4: Gene identity numbers for P. trichocarpa (Potri) and P. tremula (Potra). Additional Table S5: Subcellular localization of the candidate KTIs. Additional Table S6: Description of transformed and surviving mutant lines of Kunitz Trypsin Inhibitor in Populus x canescens. Additional Table S7: Consequences of CRISPR-Cas12a editing events observed in mutant lines. Additional Table S8: Gas exchange and growth of wildtype and transgenic poplar lines.

Additional file 2.

## Data Availability

Data generated or analysed during this study are included in this published article and its Additional information files. Materials are available upon request.
