# Supplementary material for: Divergent functions of three Kunitz trypsin inhibitor (KTI) proteins in herbivore defense in poplar
Source: BMC Plant Biol. 2025 Dec 19;26:153. doi: 10.1186/s12870-025-07955-z (PMC12837601; doi:10.1186/s12870-025-07955-z)
Supplement: Supplementary file 1 — Additional file 1: Additional Figure S1: Phylogenetic tree of putative Kunitz Trypsin Inhibitors in Populus trichocarpa and the haplotype sequences of P. tremula and P. alba. Additional Figure S2: Multiple sequence alignment of amino acids of the three candidate Kunitz Trypsin Inhibitor proteins. Additional Figure S3: Linear curves for primer efficiencies. Additional Figure S4: a) KTI transcript levels of P. x canescens plantlets grown and wounded under sterile conditions, b) KTI (Potri019G08220, KTI_8220) transcript levels of greenhouse grown poplars after exposure to methyl-jasmonate (meJA), ACC or BTH. Additional Figure S5: Transcript abundances of KTI_400, KTI_600 and KTI_53200 over-expressed under the 35S promoter. Additional Figure S6: Twelve-week-old Kunitz Trypsin Inhibitor mutant lines grown under greenhouse conditions. Additional Figure S7: Representative photographs of Kunitz Trypsin Inhibitor poplar mutant lines under constant feeding of Helicoverpa armigera larvae. Additional Table S1: Data set for potential KTIs extracted from Kaling et al. 2018. Additional Table S2: List of all primer sets used for cloning and standard PCR. Additional Table S3: List of primers for RT-qPCR. Additional Table S4: Gene identity numbers for P. trichocarpa (Potri) and P. tremula (Potra). Additional Table S5: Subcellular localization of the candidate KTIs. Additional Table S6: Description of transformed and surviving mutant lines of Kunitz Trypsin Inhibitor in Populus x canescens. Additional Table S7: Consequences of CRISPR-Cas12a editing events observed in mutant lines. Additional Table S8: Gas exchange and growth of wildtype and transgenic poplar lines. [file 12870_2025_7955_MOESM1_ESM.pdf]

Additional Materials

for

Divergent functions of three Kunitz trypsin inhibitor (KTI) proteins in herbivore defense in poplar

Ishani S. Das, Qianqian Shi, Steven Dreischhoff, Andrea Polle

Additional Figures
Additional Figure S1: Phylogenetic tree of putative Kunitz Trypsin Inhibitors in *Populus trichocarpa* and the haplotype sequences of *P. tremula* and *P. alba*.
Additional Figure S2: Multiple sequence alignment of amino acids of the three candidate Kunitz Trypsin Inhibitor proteins
Additional Figure S3: Curves for primer efficiencies.
Additional Figure S4: a) *KTI* transcript levels of *P.* x *canescens* plantlets grown and wounded under sterile conditions, b) *KTI* (Potri019G08220, KTI_8220) transcript levels of greenhouse grown poplars after exposure to meJA, ACC or BTH.
Additional Figure S5: Transcript abundances of *KTI_400, KTI_600* and *KTI_53200* over-expressed under the p*35S* promoter.
Additional Figure S6: Twelve-week-old Kunitz Trypsin Inhibitor mutant lines grown under greenhouse conditions.
Additional Figure S7: Representative photographs of Kunitz Trypsin Inhibitor poplar mutant lines under constant feeding of *Helicoverpa armigera* larvae.

Additional Tables
Additional Table S1: Data set for potential KTIs extracted from Kaling et al. 2018.
Additional Table S2: List of all primer sets used for cloning and standard PCR
Additional Table S3: List of primers for RT-qPCR
Additional Table S4: Gene identity numbers for *P. trichocarpa* (Potri) and *P. tremula* (Potra)
Additional Table S5: Subcellular localization of the candidate KTIs
Additional Table S6: Description of transformed and surviving mutant lines of *Kunitz Trypsin Inhibitor* in *Populus* x *canescens*
Additional Table S7: Consequences of CRISPR-Cas12a editing events observed in mutant lines.
Additional Table S8: Gas exchange and growth of wildtype and transgenic poplar lines

Additional Methods

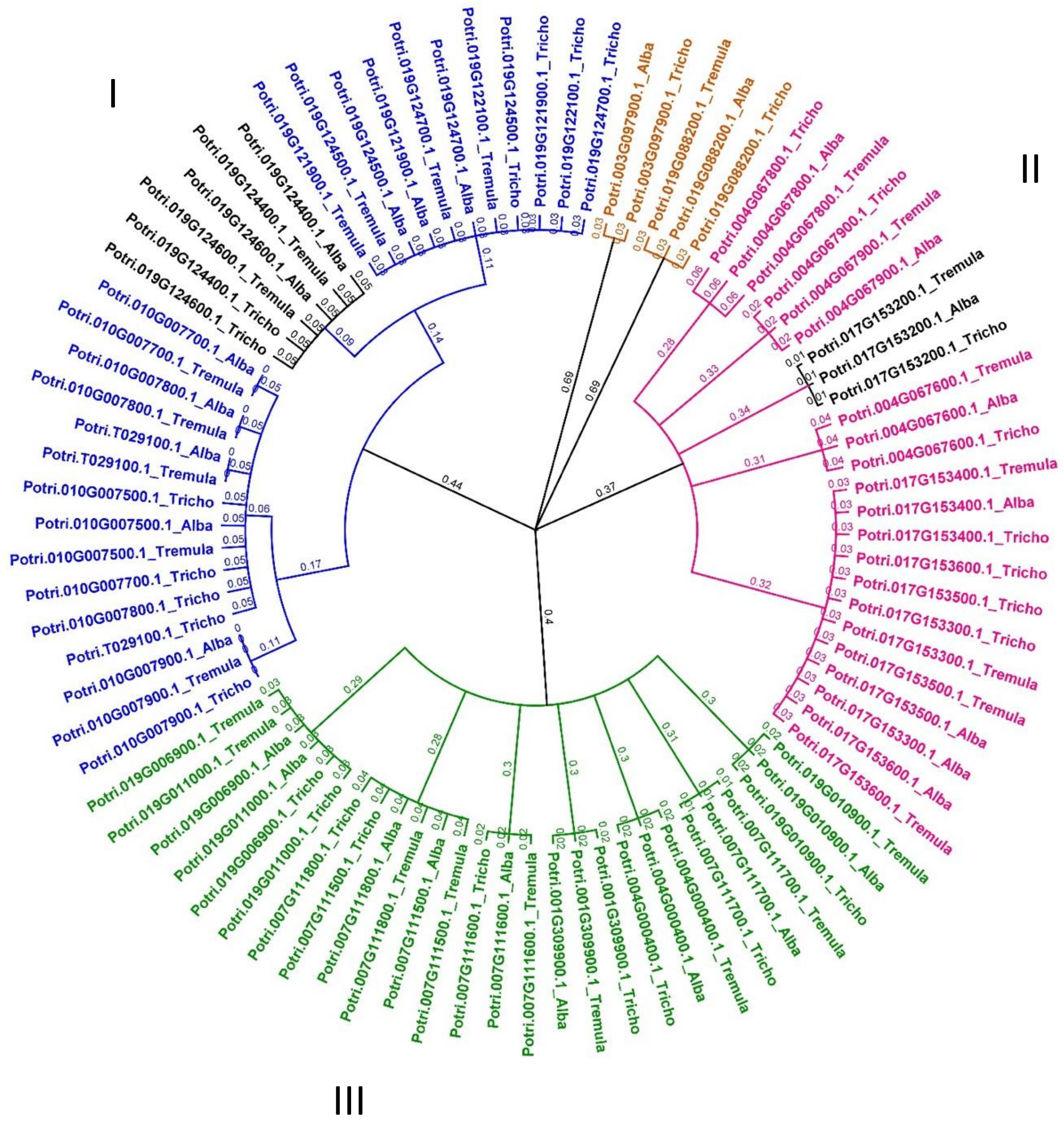

Additional Figure S1: Phylogenetic tree of putative Kunitz Trypsin Inhibitors in *Populus trichocarpa* and the haplotype sequences of *P. tremula* and *P. alba*. Unrooted UPGMA tree was generated with full-length amino acid sequences*.* sPta717. *P.* x *canescens* is a hybrid of *Populus tremula* and *Populus alba;* thus, the whole-genome sequence of *P.* x *canescens* comprises the haplotype sequences to its parents. Suffix "_Alba" or "_Tremula" denoted respective sequences from the crossing parents and "_Tricho" for the *P. trichocarpa* genome. Jukes-Cantor genetic distance model was applied with the consensus method: strict greedy clustering (Bootstrap n = 1000) in Geneious Prime (Biomatters, Ltd., Auckland, New Zealand). Colored branches denote specific clades represented as I-III. Nodes colored orange represent the KTI with the least similarity to the other KTI, and black nodes denote candidate genes in this study. Numbers denote substitutions per site used for the pairwise alignment.

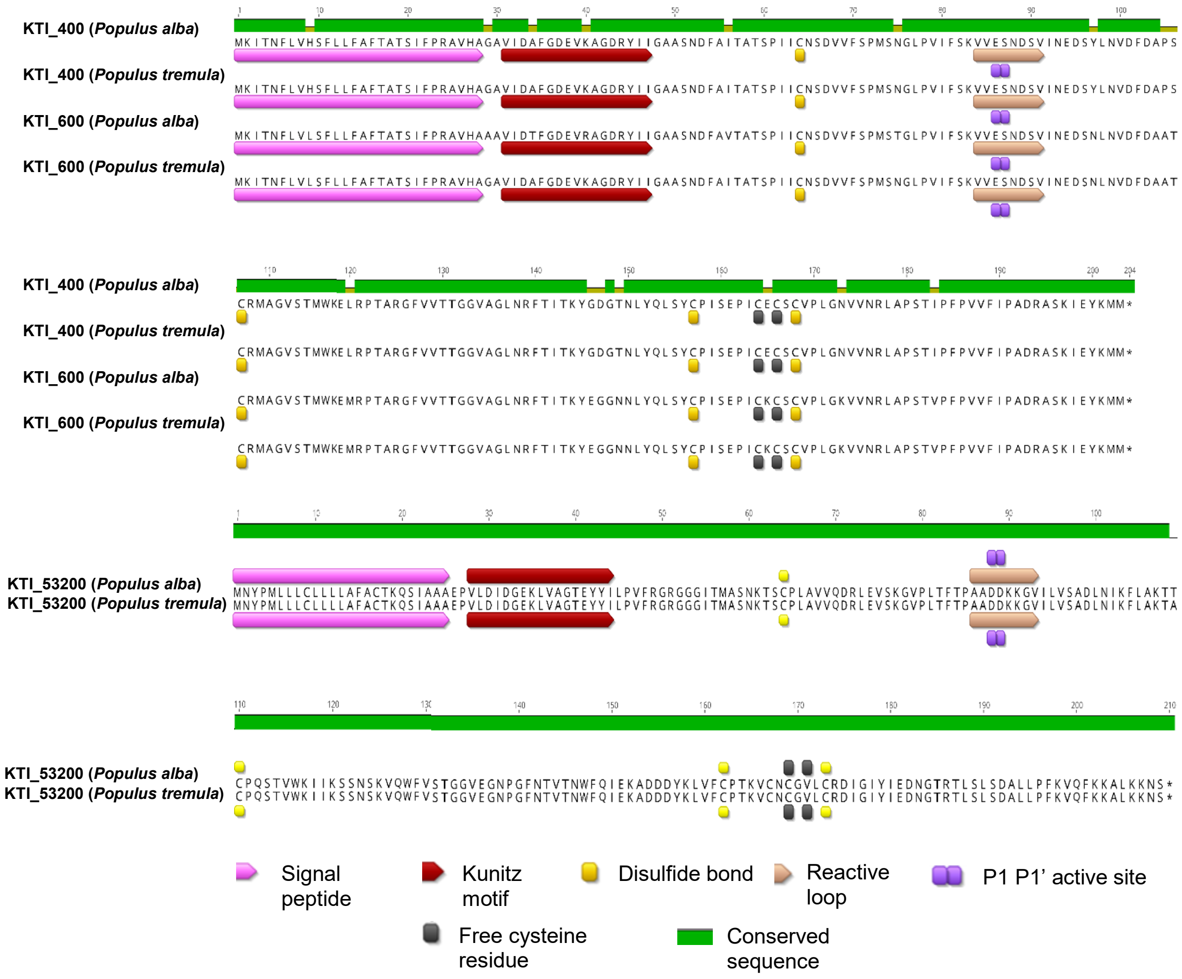

Additional Figure S2: Multiple sequence alignment of amino acids of the three candidate Kunitz Trypsin Inhibitor proteins. *Populus* x *canescens* is a hybrid of *Populus tremula* and *Populus alba;* thus, version 2 of sPta717 *P.* x *canescens* comprises the haplotype whole-genome sequences of both parents. Multiple sequence alignment performed in Clustal Omega 1.2.2 embedded in Geneious Prime (Biomatters, Ltd., Auckland, New Zealand). Kunitz motif and cysteine residues along with signal peptide, reactive loop, P1 P1' active sites, and free cysteine residues characteristic to KTIs were annotated.

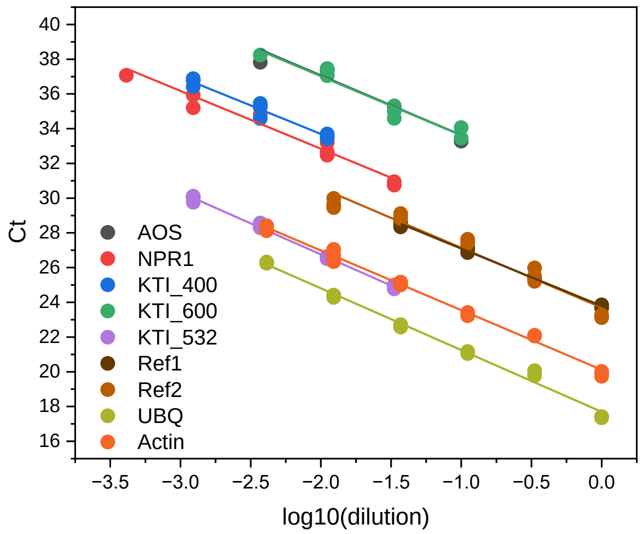

Additional Figure S3: Curves for primer efficiencies. Log10 of the dilution of the RNA extract was plotted against the Ct values and fitted by a linear model. Pearson´s R values for the curves varied from -0.98 to -0.99.

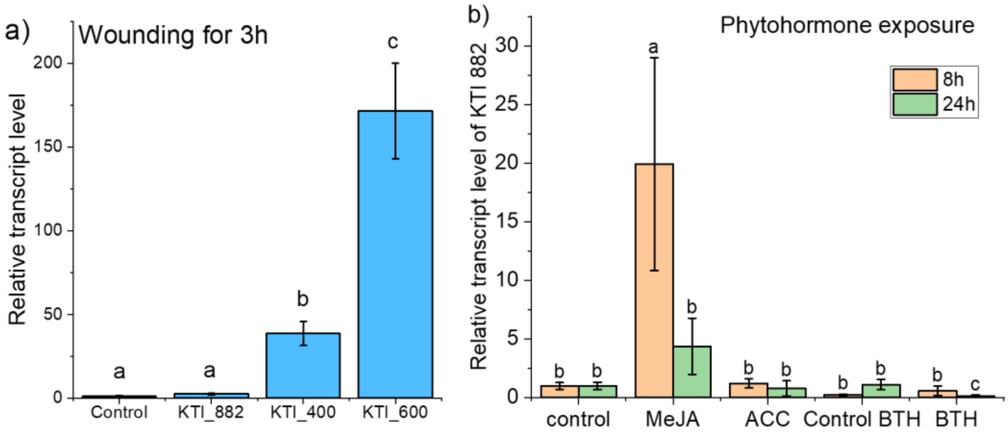

Additional Figure S4: a) KTI transcript levels of *P.* x *canescens* plantlets grown and wounded under sterile conditions, b) KTI_Potri019G08220 (KTI_8220) transcript levels of greenhouse grown poplars after exposure to methyl-jasmonate (meJA), ACC or BTH. Leaves were harvested after 3h in the wounding experiments and after 8h and 24h in the phytohormone experiments post treatment. N = 4 ± SD biological replicates per treatment. Different letters indicate significant differences at $p < 0.05$ (ANOVA, Tukey post-hoc test).

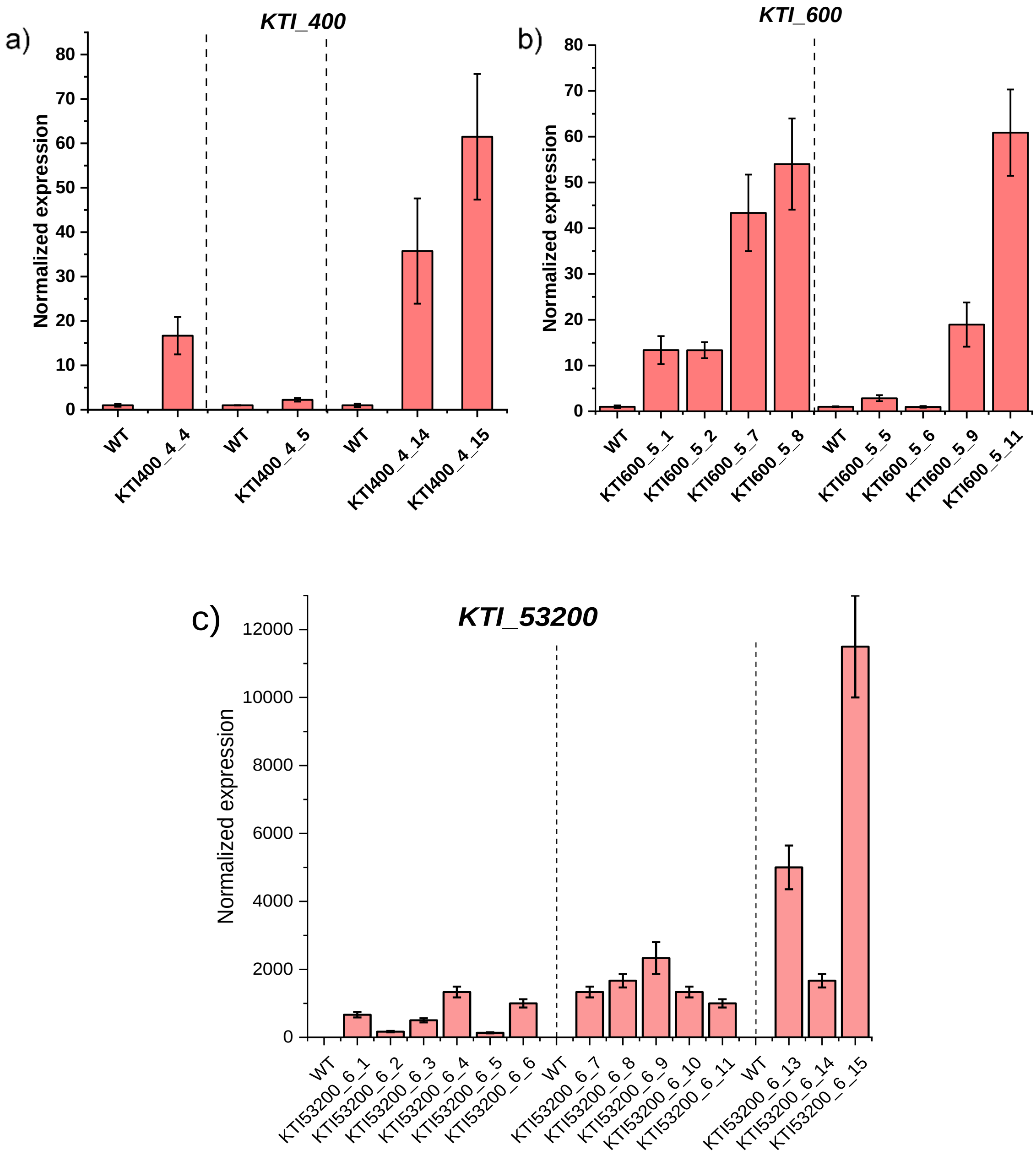

Additional Figure S5: Candidate *Kunitz Trypsin Inhibitor* genes over-expressed under the *35S* promoter. Transcript abundances of the independently transformed lines with the genes a) *KTI_400,* b) *KTI_600* and c) *KTI_53200* were tested. Transcript abundances were normalized to the reference genes, *ACTIN* and *UBIQUITIN* and were tested in comparison to the wild-type (WT) plants. Dotted lines refer to distinct experimental sets of tested plants and thus, the corresponding WT from the same experiment was compared to the mutant lines within these sets. Genotyping was performed on a single plant from each line. Error bars denote the standard deviation across three technical replicates used for RT-qPCR.

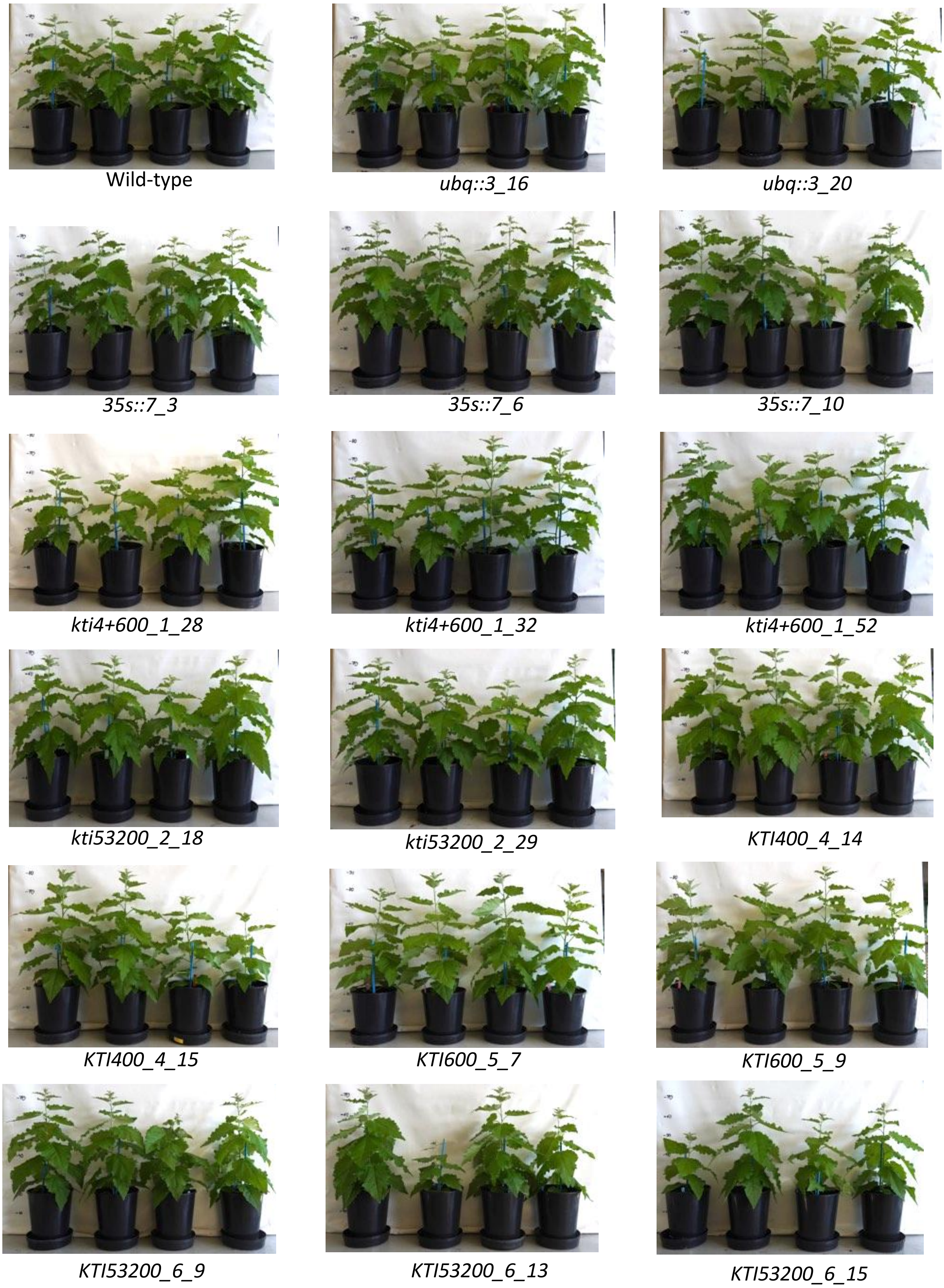

Additional Figure S6: Twelve-week-old Kunitz Trypsin Inhibitor mutant lines grown under greenhouse conditions. The panels show four biological replicates. WT, Wild type. Please, note that not all lines were available at the same time. Therefore, in some experiments, different sets of plants were used.

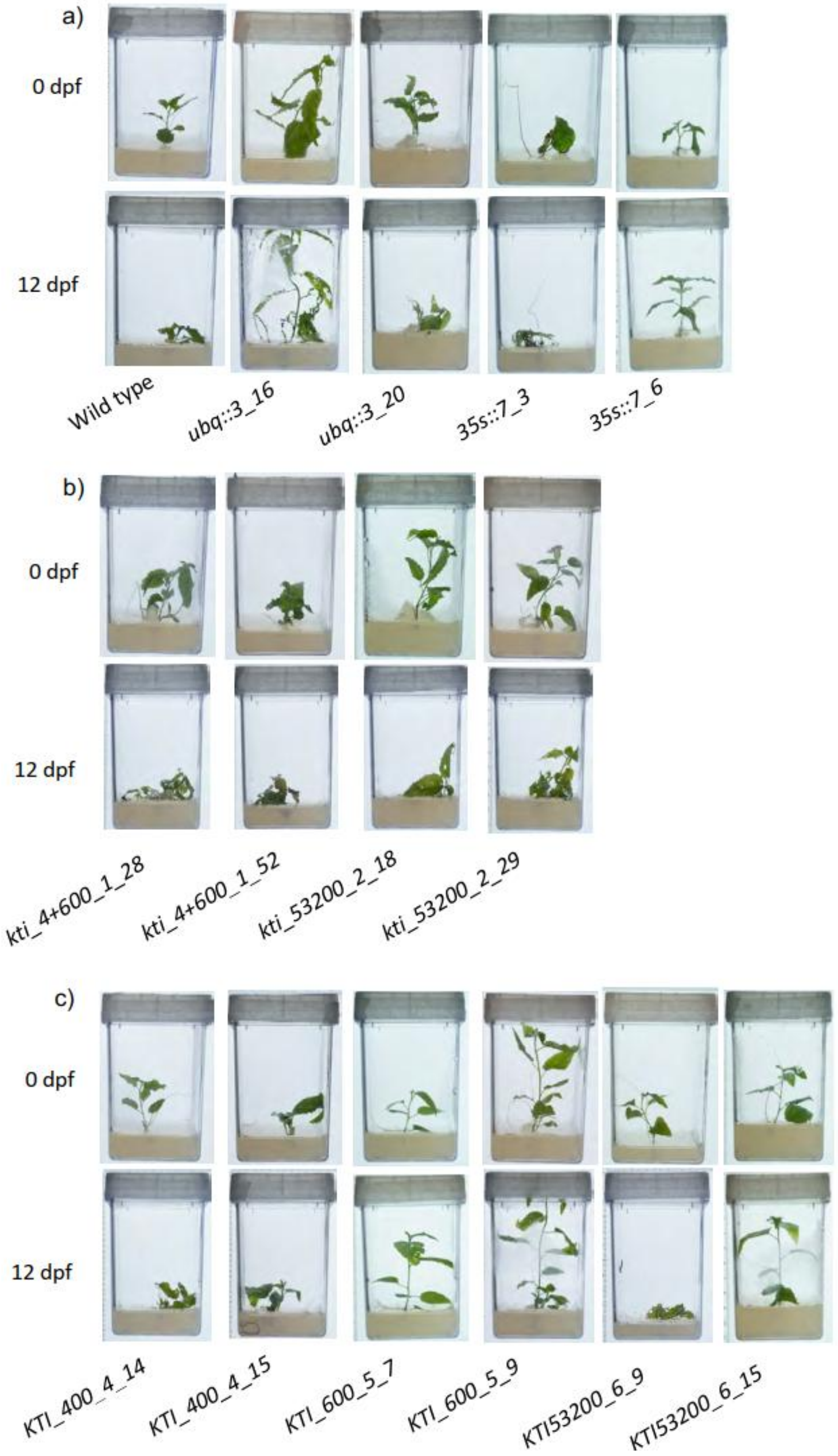

Additional Figure S7: Representative photographs of Kunitz Trypsin Inhibitor poplar mutant lines under constant feeding of *Helicoverpa armigera* larvae. Photos were taken at 0 dpf (left photograph in the panel) and 12 dpf (right photograph in the panel). dpf, days post-feeding; WT, Wildtype

Additional Table S1: Data set for potential KTIs extracted from Kaling et al. 2018. Mycorrhizal (MC) and non-mycorrhizal (NC) poplars were exposed outdoors in cages to *Chrysomela populi* (MB, NB). First fully expanded leaves from the top were harvested and used for RNAseq analyses.

	NC		MC		NB		MB		Padj	Padj	Padj	Padj
Potri	Mean nc	se	Mean m	se	Mean nc	se	Mean m	se	MC_NC	MB_MC (Padj)	NB_NC	MB_NB
Potri.004G067800	12	1	11	4	694	297	913	430	ns	3.79E-109	1.74E-96	ns
Potri.004G067900	15	3	14	2	45	25	53	17	ns	1.33E-07	3.46E-06	ns
Potri.010G007500	6	1	7	2	15	6	9	2	ns	ns	0.021203	ns
Potri.010G007700	14	5	6	3	71	25	80	22	ns	3.04E-24	1.15E-13	ns
Potri.010G007800	18	4	13	1	81	19	92	14	ns	1.65E-18	2.08E-13	ns
Potri.010G007900	14	9	10	1	723	266	716	296	ns	5.66E-98	1.40E-96	ns
Potri.017G153200	31	7	40	8	225	39	301	79	ns	5.06E-31	8.13E-30	ns
Potri.019G088200	45	17	90	52	1353	349	2123	1571	0.0076	2.77E-83	1.39E-90	ns
Potri.019G121800	25	20	8	1	824	225	726	130	0.0145	6.96E-105	7.68E-88	ns
Potri.019G121900	12	2	9	3	185	63	197	65	ns	1.70E-46	2.26E-42	ns
Potri.019G122100	10	2	7	0	201	70	199	71	ns	1.27E-51	1.80E-47	ns
Potri.019G124400	767	781	191	77	14394	4035	14900	3308	2.34E-15	1.98E-148	3.10E-88	ns
Potri.019G124500	10	0	11	3	67	37	62	16	ns	9.17E-13	8.38E-17	ns
Potri.019G124600	435	415	147	58	9992	2701	9780	2275	2.66E-08	8.94E-139	1.74E-96	ns
Potri.019G124700	29	14	22	6	683	215	683	230	ns	1.82E-79	1.65E-74	ns
Potri.T029100	13	2	12	2	12	1	13	1	ns	1.04E-19	5.01E-21	ns
Potri.004G000400	16	2	15	3	32	8	59	16	ns	3.49E-08	0.029047	ns
Potri.007G111500	23	8	19	0	171	50	209	54	ns	1.16E-35	5.22E-28	ns
Potri.007G111600	12	2	12	2	31	1	28	7	ns	0.011786	0.001315	ns
Potri.007G111700	15	3	11	2	126	42	124	32	ns	4.79E-28	3.79E-26	ns
Potri.007G111800	8	3	10	3	90	32	100	25	ns	2.83E-24	2.43E-26	ns
Potri.019G010900	11	1	8	1	36	7	45	16	ns	1.50E-10	8.82E-06	ns

Additional Table S2: List of all primer sets used for cloning and standard PCR. *Potri.019G124400* corresponds to *KTI_400, Potri.019G124600* to *KTI_600* and *Potri.017G153200* to *KTI_53200.*

Primer Name Forward	Sequence (5´→3´)	Primer Name Reverse	Sequence (5´→3´)	Amplicon size (bp)	Annealing Temperature (°C)	Purpose
KTI_6_F_2	ATATTTCCTCGTGCC GTTCATGCTG	KTI_6_R	GCATGAGCATTCACATAT GGGTTCG	444	66	*Potri.019G124400* gene and Target_400_600 specific
KTI_6_F_2	ATATTTCCTCGTGCC GTTCATGCTG	KTI_4_R_11	GGAATTATCATGTTTTAAT GCTGTGAGAG	739	64	*Potri.019G124600* (Nested PCR outer amplicon)
KTI_6_F_2	ATATTTCCTCGTGCC GTTCATGCTG	KTI_4_R_5	TTGATACGCCCGCCATTCT ACATGT	280	66	*Potri.019G124600* (Nested PCR inner amplicon) and Target_400_600_specific
KTI_5_F	GATGGTGAAAAGCTT GTAGCAGG	KTI_5_R	CCATACAGTTGATTGGGG ACATG	255	55	*Potri.017G153200* specific and Target_53200 specific
SS42	TCCCAGGATTAGAAT GATTAGG	SS143	CAAGAAAGCTGGGTCCT CAG	441	55	pChimera vector specific
SS42	TCCCAGGATTAGAAT GATTAGG	400_600_Target_R	GGCCTGATAGGGCTAGTC GCAGTGATCG	348	56	Target_400_600_integration to pChimera vector
SS42	TCCCAGGATTAGAAT GATTAGG	53200_Target_R	GGCCTTTCTTGTCGTCGG CAGCTGGTGT	348	56	Target_53200_integration to pChimera vector
SS42	TCCCAGGATTAGAAT GATTAGG	SS102	CACCATGTTATCACATCA ATCC	918 bp	56	Integration of crRNA to pDettLb-Cas12a vector; successful Gateway reaction
LbCas12a_F_2	GAAGAGAGCTGAAG ATTACAAGGG	LbCas12a_R_2	TGATCACGTTCCACTCGC CG	953	55	pDett_LbCas12a T-DNA specific
Spec_F_2	CCAGTCGGGCGGCG AGTTCC	Spec_R_2	ACGCAGCAGGGCAGTCG CCC	576	60	pDett_LbCas12a vector backbone specific

pDONR201_F	TCGCGTTAACGCTAGCATGGATCTC	pDONR201_R	GTAACATCAGAGATTTTGAGACAC	2,490	58	Amplification of pDONR vector flanking the attachment sites
29-Pro35S_Seq	GATGTGATATCTCCACTGACG	28-pK7WG_R (T35S)	TTGCGGACTCTAGCATGG	1943	55	pK7WG2 T-DNA specific
400_600_OE_F	GGGGACAAGTTTGTACAAAAAAGCAGGCTTAAAAATGAAGATCACTAACTTTCTAGTGC	400_600_OE_R	GGGGACCACTTTGTACAAGAAAGCTGGGTATTAAGCGTAATCTGGAACATCGTATGGGTACATCATTTTATACTCGATTTTAGAAGC	600	58	*Potri.019G124400* and *Potri.019G124600* cDNA amplification along with an adapter for pDONR201 vector
53200_OE_F	GGGGACAAGTTTGTACAAAAAAGCAGGCTTAAAAATGAATTATCCAATGTTATTGYTGTGC	53200_OE_R	GGGGACCACTTTGTACAAGAAAGCTGGGTATTAAGCGTAATCTGGAACATCGTATGGGTACGAGTTTTTCTTCAAAGCTTTCTTG	773	58	*Potri.017G153200* cDNA amplification along with an adapter for pDONR201 vector

Additional Table S3: List of primers, their annealing temperature, and their primer efficiencies for RT-qPCR.

Primer Name Forward	Sequence (5' → 3')	Primer Name Reverse	Sequence (5' → 3')	Amplicon size (bp)	Annealing Temp(°C)	Primer Efficiency (%)	Dilution for qRT-PCR[1]	Targeted gene ID
Actin9_F	TGGTGGTTCCACTATGTTCC	Actin9_R	TGGAAATCCACATCTGCTGG	176	50	99	1 to 90	*Potri.001G309500*
AOS_2_F	GTCTAAAAGAAAACATGTCC	AlOxS_2_R	AAATAAAACAGAAACCACAC	68	50	102	1 to 90	*Potra002586g19438*
UBQ_L_F	TGAGGCTTAGGGGAGGAACT	UBQ_L_R	TGT AGT CGC GAG CTG TCT	195	55	98	1 to 30	*Potri.005G198700*
NPR1_2_F	AGCCTTTTACAAACCCAGAAATTGC	NPR1_2_R	TAAAATGCATCTGTGAACTGGAACC	152	55	94	1 to 30	*Potri.006G148100*
KTI_4_F_2	GACACCTTCGGTGATGAGGTTAAAG	KTI_4_R_4	GTCCACATTCAGATTACTGTCTTCA	204	55	93	1 to 30	*Potri.019G124600*
KTI_3_F	ATGAAGATGACTAACTTTCTAGTGCA	KTI_3_R_2	GTCCACATTAAGATTACTGTCTTCG	300	50	102	1 to 90	*Potri.019G124400*
KTI_5_F	GATGGTGAAAAGCTTGTAGCAGG	KTI_5_R_2	CCTTTCTTGTCGTCGGCA	182	50	103	1 to 9	*Potri.017G153200*
A_Ref2_F	ATCGTTCCAAGTCAAGTATGTG	A_Ref2_R	TCAAGGGAGCAACTTTACAG	93	50	101	1 to 9	*Potri.015G001600*
C_Ref1_F	GCAATGTGAGGAGTTTAGGG	C_Ref1_R	TATTAAATGTCTGTGCTGTAGTGTG	86	50	95	1 to 9	*Potri.012G141400*
PtKTIup1_RT_F	GTGTCCAACTACAAGCTCCT	PtKTIup1_RT_R	TCAAAGCCAGGTATCTAATCCC	117	50	98	1 to 9	*Potri.019G088200*
PtERF1_RT_F	TTCCAGAGCAATTCACTTCCT	PtERF1_RT_R	CACTTCCTCTTCCTTAATTCCAC	136	50	90	1 to 9	*Potri.T131500*

[1]The dilution of cDNA to be performed on stock cDNA synthesized by the RevertAid first strand cDNA synthesis kit (Thermo Fisher Scientific).

Additional Table S4: Gene identity numbers for *P. trichocarpa* (Potri) and *P. tremula* (Potra). The annotations were downloaded from Plantgenie (https://plantgenie.org/, accessed: 25.05.2025). The gene IDs are associated with GO:0004866: endopeptidase inhibitor activity and have the pfam_description: PF00197-Trypsin and protease inhibitor.

Potri_ID	chromosome name	atg_id	Potra_gene_id	description
Potri.001G309900	Chr01	AT1G17860	Potra2n1c2691	similar to truncated Kunitz trypsin inhibitor. [ORG:Glycine max]; [co-ortholog (1of6) of AAK20290]
Potri.003G097900	Chr03		Potra2n3c7636	
Potri.004G000400	Chr04	AT1G73260	Potra2n4c8413	similar to truncated Kunitz trypsin inhibitor. [ORG:Glycine max]; [co-ortholog (6of6) of AAK20290]
Potri.004G067600	Chr04	AT1G17860	Potra2n4c9034	
Potri.004G067800	Chr04	AT1G17860	Potra2n4c9037	similar to trypsin protein inhibitor 3. [ORG:Cicer arietinum]; [co-ortholog (1of3) of S19190, BAD04942, BAD04939, JC7311, AAB21123, B45588, P09941, P25272, P83594, JQ0968, AAB23464, 1AVX_B, TISYB, AAB26177, CAH61462, P32733, P83051, AAA32618, AAT45474]
Potri.004G067900	Chr04	AT1G17860	Potra2n4c9039	similar to trypsin protein inhibitor 3. [ORG:Cicer arietinum]; [co-ortholog (2of3) of S19190, BAD04942, BAD04939, JC7311, AAB21123, B45588, P09941, P25272, P83594, JQ0968, AAB23464, 1AVX_B, TISYB, AAB26177, CAH61462, P32733, P83051, AAA32618, AAT45474]
Potri.007G111500	Chr07	AT1G73260	Potra2n416s35592	
Potri.007G111600	Chr07	AT1G17860	Potra2n416s35593	
Potri.007G111700	Chr07		Potra2n416s35593	similar to truncated Kunitz trypsin inhibitor. [ORG:Glycine max]; [co-ortholog (4of6) of AAK20290]
Potri.007G111800	Chr07	AT1G73260	Potra2n416s35592	
Potri.010G007500	Chr10		Potra2n156s34746	
Potri.010G007700	Chr10		Potra2n156s34746	
Potri.010G007800	Chr10		Potra2n156s34746	
Potri.010G007900	Chr10		Potra2n156s34746	
Potri.017G153200	Chr17	AT1G17860	Potra2n17c30597	similar to trypsin protein inhibitor 3. [ORG:Cicer arietinum]; [co-ortholog (3of3) of S19190, BAD04942, BAD04939, JC7311, AAB21123, B45588, P09941, P25272, P83594, JQ0968, AAB23464, 1AVX_B, TISYB, AAB26177, CAH61462, P32733, P83051, AAA32618, AAT45474]
Potri.017G153300	Chr17	AT1G17860	Potra2n17c30594	

Potri.017G153400	Chr17	AT1G17860	Potra2n17c30594	
Potri.017G153500	Chr17	AT1G17860	Potra2n17c30594	
Potri.017G153600	Chr17	AT1G17860	Potra2n17c30594	
Potri.019G006900	Chr19	AT1G73260	Potra2n19c34329	
Potri.019G010900	Chr19	AT1G17860	Potra2n19c34330	
Potri.019G011000	Chr19	AT1G73260	Potra2n19c34325	similar to truncated Kunitz trypsin inhibitor. [ORG:Glycine max]; [co-ortholog (2of6) of AAK20290]
Potri.019G088200	Chr19	AT1G17860	Potra2n19c33577	
Potri.019G121900	Chr19		Potra2n19c33329	
Potri.019G122100	Chr19		Potra2n19c33329	
Potri.019G124400	Chr19		Potra2n19c33329	
Potri.019G124500	Chr19		Potra2n19c33329	
Potri.019G124600	Chr19		Potra2n19c33329	
Potri.019G124700	Chr19		Potra2n19c33329	
Potri.T029100	scaffold_31		Potra2n156s34746	

Additional Table S5: *In-silico* prediction of the subcellular localization of the candidate KTIs according to different bioinformatics tools. The highest probabilities are highlighted in green.

	Potri.019G124600	Potri.019G124400	Potri.017G153200
**Membrane association#**	**Probability**	**Probability**	**Probability**
**Peripheral**	0.238	0.243	0.124
**Transmembrane**	0.042	0.04	0.048
**Lipid anchor**	0.039	0.039	0.052
**Soluble**	0.891	0.895	0.898
**Localization#**	**Probability**	**Probability**	**Probability**
**Predicted signals**	Signal peptide	Signal peptide	Signal peptide
**Cytoplasm**	0.0706	0.0605	0.1039
**Nucleus**	0.1476	0.1699	0.1532
**Extracellular**	0.7673	0.7797	0.8694
**Cell membrane**	0.0951	0.0838	0.1277
**Mitochondrion**	0.0098	0.0102	0.0075
**Plastid**	0.1589	0.2342	0.015
**Endoplasmic reticulum**	0.3958	0.3046	0.2826
**Lysosome/Vacuole**	0.2562	0.2135	0.244
**Golgi apparatus**	0.0709	0.0569	0.0706
**Peroxisome**	0.0023	0.0038	0.0031

https://services.healthtech.dtu.dk/services/DeepLoc-2.1/

**Scores für Localisation***	Potri.019G124600	Potri.019G124400	Potri.017G153200
**Cytoplasm**			1
**Nucleus**			
**Extracellular**	4	4	11
**Cell membrane**			
**Mitochondrion**		1	1
**Plastid**	8	5	1
**Endoplasmic reticulum**	1.5		
**Lysosome/Vacuole**	1.5	4	
**Golgi apparatus**		1	

*https://wolfpsort.hgc.jp/

Additional Table S6: Description of transformed and surviving mutant lines of *Kunitz Trypsin Inhibitor* in *Populus* x *canescens.*

Line number	Vector used	Purpose	Gene target	Deletion (bp) [1]
1_8	pDett-Lb-Cas12a_400_600	loss-of-function	*Potri.019G124400* and *Potri.019G124600*	8
1_22	pDett-Lb-Cas12a_400_600	loss-of-function	*Potri.019G124400* and *Potri.019G124600*	6
1_28	pDett-Lb-Cas12a_400_600	loss-of-function	*Potri.019G124400* and *Potri.019G124600*	50
1_32	pDett-Lb-Cas12a_400_600	loss-of-function	*Potri.019G124400* and *Potri.019G124600*	14
1_37	pDett-Lb-Cas12a_400_600	loss-of-function	*Potri.019G124400* and *Potri.019G124600*	10
1_41	pDett-Lb-Cas12a_400_600	loss-of-function	*Potri.019G124400* and *Potri.019G124600*	9
1_52	pDett-Lb-Cas12a_400_600	loss-of-function	*Potri.019G124400* and *Potri.019G124600*	50
1_54	pDett-Lb-Cas12a_400_600	loss-of-function	*Potri.019G124400* and *Potri.019G124600*	50
2_18	pDett-Lb-Cas12a_53200	loss-of-function	*Potri.017G153200*	7
2_29	pDett-Lb-Cas12a_53200	loss-of-function	*Potri.017G153200*	10
2_43	pDett-Lb-Cas12a_53200	loss-of-function	*Potri.017G153200*	5
2_45	pDett-Lb-Cas12a_53200	loss-of-function	*Potri.017G153200*	5
3_7	pDett_LbCas12a_pEn-RZ-Lb_chimera	Ubiquitin_Promoter _EmptyVector	Empty vector control	N/A
3_15	pDett_LbCas12a_pEn-RZ-Lb_chimera	Ubiquitin_Promoter _EmptyVector	Empty vector control	N/A
3_16	pDett_LbCas12a_pEn-RZ-Lb_chimera	Ubiquitin_Promoter _EmptyVector	Empty vector control	N/A
3_20	pDett_LbCas12a_pEn-RZ-Lb_chimera	Ubiquitin_Promoter _EmptyVector	Empty vector control	N/A

[1] All deletions were induced in exons. Length of deletions determined via Sanger sequencing.

Additional Table S7: Consequences of CRISPR-Cas12a editing events observed in mutant lines.

Plant ID	Gene	CRISPR-Cas12a system	Predicted truncated amino acid length	No. premature stop codons
WT KTI_400*	*KTI_400*	MKITNFLVHSFLLFAFTATSIFPRAV HAGAVIDAFGDEVKAGDRYIIGAAS NDFAITATSPIICNSDVVFSPMSNGL PVIFSKVVESNDSVINEDSYLNVDF DAPSCRMAGVSTMWKIELRPTARG FVVTTGGVAGLNRFTITKYGDGTN LYQLSYCPISEPICECSCVPLGNVVN RLAPSTIPFPVVFIPADRASKIEYKM M*	204 (full-length)	
WT KTI_600*	*KTI_600*	MKITNFLVLSFLLFAFTATSIFPRAVH AAAVIDTFGDEVRAGDRYIIGAASN DFAVTATSPIICNSDVVFSPMSTGLP VIFSKVVESNDSVINEDSNLNVDFD AATCRMAGVSTMWKIEMRPTARG FVVTTGGVAGLNRFTITKYEGGNN LYQLSYCPISEPICKCSCVPLGKVVN RLAPSTVPFPVVFIPADRASKIEYK MM*	204 (full-length)	
kti4+600_1_52	*KTI_400*	MKITNFLVHSFLLFAFTATSIFPRAV HAGAVIDAFGDEVKAGDRYIIGARC CVFSDEQWTPCNIFKSCRIQRQCHQ RRQLSECGL*CTLM*DGGRLNHVE D*IEANSARIRCDHRRCCWIESVYD HQVWRWYQFVSAFLLSNFRTHM* MLMRPTRQRCQSLGSQYHPFSCCV YTSR*SF*NRV*NDV	86; 91; 102; 149; 178; 181; 185	7
	KTI_600	MKITNFLVLSFLLFAFTATSIFPRAVH AAAVIDTFGDEVRAGDRYIIGARCC VFSDEHWTPSNIFKSCRIQRQCHQR RQ*SECGL*CSHM*NGGRINHVED* NEANSARIRCDHRRCCWIESVYDH QV*RW**FVSAFLLSNFGTHM*ML MRPTRQSCQSLGSQYRPFSSCVYTS R*SF*NRV*NDV	81; 87; 92; 103; 130; 133; 134; 149; 178; 181; 185	11

kti4+600_1_28	*KTI_400*	MKITNFLVHSFLLFAFTATSIFPRAV HAGAVIDAFGDEVKAGDRYIIGARC CVFSDEQWTPCNIFKSCRIQRQCHQ RRQLSECGL*CTLM*DGGRLNHVE D*IEANSARIRCDHRRCCWIESVYD HQVWRWYQFVSAFLLSNFRTHM* MLMRPTRQRCQSLGSQYHPFSCCV YTSR*SF*NRV*NDV	86; 91; 102; 149; 178; 181; 185	7
	KTI_600	MKITNFLVLSFLLFAFTATSIFPRAVH AAAVIDTFGDEVRAGDRYIIGARCC VFSDEHWTPSNIFKSCRIQRQCHQR RQ*SECGL*CSHM*NGGRINHVED* NEANSARIRCDHRRCCWIESVYDH QV*RW**FVSAFLLSNFGTHM*ML MRPTRQSCQSLGSQYRPFSSCVYTS R*SF*NRV*NDV	81; 87; 92; 103; 130; 133; 134; 149; 178; 181; 185	11
WT KTI_53200*	*KTI_53200*	MNYPMLLLCLLLLAFACTKQSIAA AEPVLDIDGEKLVAGTEYYILPVFR GRGGGITMASNKTSCPLAVVQDRL EVSKGVPLTFTPAADDKKGVILVSA DLNIKFLAKTTCPQSTVWKIIKSSNS KVQWFVSTGGVEGNPGFNTVTNW FQIEKADDDYKLVFCPTKVCNCGV LCRDIGIYIEDNGTRTLSLSDALLPF KVQFKKALKKNS*	210 (full-length)	
kti53200_2_18	*KTI_53200*	MNYPMLLLCLLLLAFACTKQSIAA AEPVLDIDGEKLVAGTEYYILPVFR GRGGGITMASNKTSCPLAVVQDRL EVSKGVPLTFTPAADKV*SLFLLILT SSF*RRQHVPNQLYGRL*SLRTRRY NGLCQLVGLKEILVLIR*PTGSRLRK LMMTTSLFSVLLKFVTVEFYAGILG FILRIMGLEHCLSVMHYYLSKSSSR KL*RKTR	92; 104; 118; 143; 204	5
kti53200_2_29	*KTI_53200*	MNYPMLLLCLLLLAFACTKQSIAA AEPVLDIDGEKLVAGTEYYILPVFR GRGGGITMASNKTSCPLAVVQDRL EVSKGVPLTFTPAADE*SLFLLILTSS F*RRQHVPNQLYGRL*SLRTRRYNG LCQLVGLKEILVLIR*PTGSRLRKLM MTTSLFSVLLKFVTVEFYAGILGFIL RIMGLEHCLSVMHYYLSKSSSRKL* RKTR	91; 103; 117; 142; 203	5

*WT amino acid sequences from *KTI_400, KTI_600* and *KTI_53200.*
Red-colored amino acid residue denotes signal peptide; green denotes Kunitz. Motif; blue reactive loop and yellow disulfide bridge. *denotes the stop codon.

Additional Table S8: Gas exchange and growth of wildtype and transgenic poplar lines (*P*. x *canescens*). N = 4 ± SE, WT: n= 8. Different letters indicate significant differences of means ($p < 0.05$, Tukey test).

Line	Count	Transpiration mol/m²		SE	Gs mmol/m²s1	SE		Stem height cm		SE mm	Stem diameter		SE		Root-to-Shoot	SE
WT	8	4.77	0.18	AB	0.228	0.013	A	57.31	2.34	A	6.88	0.22	A	0.77	0.12	DE
OXEV_3	4	4.74	0.14	AB	0.218	0.011	A	63.63	1.53	AB	6.92	0.18	A	0.48	0.02	ABCDE
OX532_8	4	4.32	0.24	AB	0.198	0.008	A	68.68	2.69	AB	7.45	0.32	A	0.41	0.02	ABC
OX532_4	4	4.25	0.19	AB	0.188	0.010	A	66.03	1.24	AB	7.80	0.24	A	0.45	0.02	ABCD
OX532_10	4	4.36	0.08	AB	0.200	0.007	A	60.55	2.76	AB	6.98	0.67	A	0.37	0.04	AB
OX400_5	4	4.18	0.43	AB	0.193	0.027	A	64.88	0.63	AB	6.55	0.29	A	0.52	0.03	ABCDE
OX400_4	4	3.53	0.22	A	0.160	0.011	A	63.10	1.73	AB	6.80	0.16	A	0.45	0.04	ABCD
OX400_15	4	5.21	0.38	B	0.243	0.023	A	55.43	2.75	A	6.48	0.31	A	0.33	0.04	A
OX400_14	4	3.98	0.30	AB	0.178	0.019	A	59.80	5.52	AB	7.25	0.28	A	0.40	0.03	ABC
kti532_43	4	4.00	0.18	AB	0.180	0.004	A	65.60	2.23	AB	7.16	0.36	A	0.44	0.04	ABC
kti532_29	4	3.67	0.33	A	0.160	0.024	A	66.13	3.76	AB	7.31	0.35	A	0.47	0.03	ABCDE
kti532_18	4	3.87	0.23	AB	0.168	0.015	A	64.98	0.80	AB	6.98	0.07	A	0.51	0.07	ABCDE
kti400_41	4	3.68	0.37	A	0.158	0.022	A	68.18	2.86	AB	7.79	0.25	A	0.73	0.11	BCDE
kti400_32	4	3.60	0.38	A	0.160	0.017	A	71.43	1.88	B	7.82	0.36	A	0.85	0.05	E
kti400_28	4	4.33	0.29	AB	0.193	0.020	A	65.60	2.16	AB	7.91	0.32	A	0.78	0.04	CDE
koev_16	4	3.94	0.33	AB	0.178	0.018	A	68.73	2.47	AB	7.99	0.41	A	0.48	0.03	ABCDE
koev_15	4	3.82	0.29	AB	0.170	0.019	A	70.50	0.74	B	7.66	0.19	A	0.41	0.04	ABC
Total	72	4.13	0.11		0.186	0.006		64.74	1.07		7.28	0.12		0.52	0.04	
